# Clinical and Economic Outcomes Associated with Structured Preoperative Anemia Management in Elective Cardiac Surgery: A Patient Blood Management Cohort Study

**DOI:** 10.3390/jcm15187150

**Published:** 2026-09-15

**Authors:** Henrique Coelho, Bruno Daniel Carneiro, Ana Borges, Diana Paupério, Fernando Silva, Maria Inês Barbosa, Marta Correia, Pedro Miguel Rodrigues

**Affiliations:** 1 CBQF—Centro de Biotecnologia e Química Fina—Laboratório Associado, Escola Superior de Biotecnologia, Universidade Católica Portuguesa, 4169-005 Porto, Portugal; mibarbosa@ucp.pt (M.I.B.); mmcorreia@ucp.pt (M.C.); pmrodrigues@ucp.pt (P.M.R.); 2 Serviço de Hematologia, Unidade Local de Saúde de Vila Nova de Gaia e Espinho, 4434-502 Vila Nova de Gaia, Portugal; bcarneiro@med.up.pt (B.D.C.); ana.m.borges@ulsb.min-saude.pt (A.B.); 3 Departamento de Ciências Médicas, Universidade de Aveiro, 3810-193 Aveiro, Portugal; diana.pauperio@ulsge.min-saude.pt; 4 Unidade de Biologia Experimental, Departamento de Biomedicina, Faculdade de Medicina da Universidade do Porto, 4200-319 Porto, Portugal; 70836@ulsra.min-saude.pt; 5 Serviço de Anestesiologia, Unidade Local de Saúde de Vila Nova de Gaia e Espinho, 4434-502 Vila Nova de Gaia, Portugal; 6 Serviço de Hematologia, Unidade Local de Saúde da Região de Aveiro, 3814-501 Aveiro, Portugal

**Keywords:** patient blood management, preoperative anemia, cardiac surgery, iron deficiency, red blood cell transfusion, hospital costs, readmission, propensity-score matching, cost-consequence analysis, safety outcomes

## Abstract

**Background/Objectives**: Preoperative anemia and iron deficiency increase transfusion risk in cardiac surgery. We evaluated outcomes associated with a Patient Blood Management (PBM) pathway and assessed the robustness of these associations to baseline imbalance and secular trends. **Methods**: This single-center retrospective cohort study compared PBM patients treated in 2019, 2022, and 2023 with historical controls from 2017–2018. Outcomes included red blood cell (RBC) transfusion, length of stay, readmission, mortality, safety events, and costs. Analyses included multivariable regression, propensity-score matching (PSM), inverse probability of treatment weighting (IPTW), and adjacent-period matching. The hospital-perspective cost-consequence analysis used 2024-unit costs, bootstrap confidence intervals (CI), and log-link models. **Results**: Among 1374 patients (764 PBM; 610 No-PBM controls), any RBC transfusion occurred in 17.3% versus 39.2%, and transfusion of >2 units occurred in 2.6% versus 15.1%. The adjusted odds ratios were 0.30 (95% CI, 0.23–0.39) and 0.14 (95% CI, 0.08–0.24), respectively. PSM yielded 519 pairs, and sensitivity analyses were concordant. The adjusted overall direct-cost ratio was 0.75 (95% CI, 0.68–0.82), with an adjusted marginal mean difference of −EUR 5196 (bootstrap 95% CI, −EUR 6965 to −EUR 3485). Recorded incidences of myocardial infarction, stroke, thromboembolism, and cumulative infection were low and did not differ significantly between cohorts; hypophosphatemia and treatment-specific adverse reactions were not systematically assessed. Adjacent-period matching did not confirm a reduction in 12-month readmission, and mortality was similar between cohorts. **Conclusions**: PBM was associated with lower transfusion rates, shorter hospitalization, and lower direct costs. Residual temporal confounding limits causal interpretation, and the economic findings do not constitute formal cost-effectiveness results.

## 1. Introduction

Preoperative anemia and iron deficiency are common, clinically relevant, and potentially modifiable conditions in patients undergoing cardiac surgery. Hemodilution during cardiopulmonary bypass, surgical bleeding, coagulopathy, and postoperative inflammation may further reduce red-cell reserve and increase exposure to allogeneic red blood cell (RBC) transfusion [1,2]. Contemporary cardiac-surgery statements and guidelines indicate that preoperative anemia affects a substantial proportion of patients referred for cardiac surgery and is associated with greater transfusion requirements, postoperative morbidity and mortality, prolonged hospitalization, and increased resource use [1]. Iron deficiency, whether absolute or functional, is among the most common and treatable causes of perioperative anemia and may also occur in patients without overt anemia [3,4,5,6,7].

RBC transfusion remains an essential and potentially life-saving intervention for patients with major bleeding, impaired oxygen delivery, or limited physiological tolerance of anemia. Nevertheless, transfusion exposure has important clinical and economic consequences. In cardiac surgery, RBC transfusion has been associated with prolonged mechanical ventilation, infectious and renal complications, and composite postoperative morbidity, with evidence of a dose-dependent relationship between the number of units transfused and adverse outcomes [8]. Contemporary practice therefore aims not to withhold transfusion when clinically indicated, but to minimize unnecessary exposure to allogeneic blood while maintaining adequate tissue oxygen delivery and patient safety.

Patient Blood Management (PBM) provides a structured framework for achieving this goal. PBM is a patient-centered, systematic, and evidence-based approach that seeks to improve outcomes by managing and preserving patients’ own blood while promoting safety and patient engagement [9]. In the perioperative setting, it is built on three complementary pillars: optimizing red-cell mass, minimizing blood loss, and improving physiological tolerance of anemia [9,10]. In cardiac surgery, international and European guidance recommend implementation through a multidisciplinary pathway involving anesthesiologists, cardiac surgeons, perfusionists, intensivists, hematologists, transfusion-medicine specialists, nurses, and laboratory professionals [1,2].

The first PBM pillar—preoperative identification and treatment of anemia and hematinic abnormalities—is especially important in elective cardiac surgery, where correctable conditions can be addressed before procedures with substantial blood-loss and transfusion risks. Current consensus increasingly supports a sex-neutral hemoglobin (Hb) threshold of <13 g/dL for defining preoperative anemia before major surgery, because traditional World Health Organization sex-specific thresholds may overlook clinically relevant anemia in women undergoing procedures with moderate-to-high expected blood loss [10,11,12]. Women with Hb concentrations of 12.0–12.9 g/dL, for example, have greater transfusion exposure than those with concentrations ≥13 g/dL [12]. Etiological assessment should include iron-status testing with ferritin and transferrin saturation and, when available, functional markers such as reticulocyte hemoglobin content, because inflammation and chronic cardiovascular disease can complicate the interpretation of ferritin alone [3,5,6].

Several studies have evaluated preoperative anemia-management strategies in cardiac surgery. Cahill et al. reported that implementation of a multidisciplinary preoperative anemia-management program in elective cardiovascular surgery was associated with higher day-of-surgery Hb, fewer RBC units transfused, shorter intensive care and hospital stay, and lower transfusion-related costs [13]. Other studies have suggested that systematic correction of anemia and iron deficiency using intravenous iron, with or without erythropoiesis-stimulating agents, may improve preoperative Hb and reduce transfusion exposure in selected cardiac-surgery patients [14,15,16]. However, the available evidence remains heterogeneous. The magnitude of benefit may depend on baseline Hb, anemia phenotype, iron status, renal function, treatment timing, surgical complexity, and institutional transfusion practice. It may also depend on whether treatment is delivered as an isolated pharmacological intervention or as part of a broader multidisciplinary PBM pathway.

The economic implications of PBM are also increasingly relevant. The hospital costs associated with perioperative anemia and transfusion extend beyond the acquisition cost of RBC units and may include intensive care and ward hospitalization, readmissions, complications, treatment logistics, and broader resource utilization. Previous analyses suggest that preoperative anemia-management programs in cardiac surgery may have favorable hospital cost consequences when reductions in transfusion exposure and length of stay are considered alongside the costs of preoperative assessment and treatment [17,18]. In Portugal, evaluations of ferric carboxymaltose within PBM programs in National Health Service hospitals have also reported favorable clinical and economic associations [19]. Nevertheless, cardiac surgery-specific real-world evidence remains limited, and economic estimates may be sensitive to the analytical perspective, institutional costing methods, and the typically right-skewed distribution of hospital costs.

Against this background, we evaluated the clinical and economic outcomes associated with a structured preoperative anemia-management pathway implemented within a PBM program for elective cardiac surgery at a Portuguese tertiary center. We assessed Hb optimization, RBC transfusion exposure and intensity, postoperative length of stay, readmission, mortality, and recorded safety outcomes. We also examined whether the observed associations persisted after adjustment for measured baseline differences and in sensitivity analyses addressing potential secular trends. The economic component was a retrospective hospital-perspective cost-consequence analysis in which direct hospital costs and clinical consequences were reported separately, without estimating quality-adjusted life-years, incremental cost-effectiveness ratios, or societal costs.

## 2. Materials and Methods

### 2.1. Study Design, Setting, and Ethics

This single-center retrospective cohort study used historical controls to evaluate clinical and economic outcomes associated with a structured preoperative anemia-management pathway within a PBM program for adults undergoing elective cardiac surgery at a Portuguese tertiary-care hospital.

In 2019, the institution established a multidisciplinary PBM team to optimize preoperative Hb concentrations in patients scheduled for elective cardiac surgery. The team comprised hematologists, anesthesiologists, cardiothoracic surgeons, transfusion-medicine specialists, nurses, and laboratory professionals. Based on multidisciplinary consensus, the institution implemented a standardized protocol covering preoperative anemia screening, etiological assessment, targeted treatment, and perioperative follow-up.

The PBM cohort included eligible patients managed through the institutional pathway in 2019, 2022, and 2023. The years 2020 and 2021 were excluded because the coronavirus disease 2019 (COVID-19) pandemic substantially disrupted elective cardiac-surgery activity. The historical control (No-PBM) cohort included all consecutive patients who underwent elective cardiac surgery in 2017–2018, before implementation of the structured pathway, and who retrospectively met the same referral and eligibility criteria as the PBM cohort.

The Ethics Committee for Health of Centro Hospitalar de Vila Nova de Gaia/Espinho, EPE, Portugal, approved the broader PBM-TIPS project, including implementation of the PBM pathway and creation of a clinical database for future PBM-related research (approval No. 196/2023-1; 24 October 2023). This secondary retrospective analysis was conducted within that approved framework and in accordance with the Declaration of Helsinki; no additional study-specific approval was required. At the PBM consultation, patients evaluated in the PBM clinic provided written informed consent for the future use of routinely collected clinical data in PBM-related research. The Ethics Committee waived individual consent for the historical No-PBM cohort because the analysis used routinely collected clinical data. All data were retrieved and processed in accordance with the approved framework and applicable data-protection requirements.

### 2.2. Study Population and Procedure Classification

Adults aged ≥18 years who underwent elective cardiac surgery during the prespecified study periods were eligible for inclusion. Eligible procedures included coronary artery bypass grafting (CABG), including minimally invasive direct coronary artery bypass (MIDCAB); aortic, mitral, or tricuspid valve repair or replacement; ascending aortic replacement; composite aortic root replacement using the Bentall procedure; surgical closure of an atrial septal defect; multivalve surgery involving two or three valves; and combined procedures, including CABG performed concomitantly with valve or aortic surgery.

Patients were excluded if they underwent urgent or emergency surgery; if the anticipated interval to surgery was <2 weeks and therefore insufficient for preoperative assessment and potential Hb optimization; if they had active malignancy; if they underwent transcatheter or endovascular procedures outside the conventional surgical PBM pathway; or if their medical records lacked data for the principal outcomes of interest.

For baseline description and propensity-score analyses, procedures were assigned to one of six mutually exclusive categories: (1) CABG-containing procedures, comprising isolated CABG, MIDCAB, and any combined procedure involving CABG; (2) aortic valve surgery, comprising aortic valve repair or replacement; (3) mitral or tricuspid valve surgery, comprising mitral valve repair or replacement and isolated tricuspid valve surgery; (4) ascending aortic surgery, comprising ascending aortic replacement and the Bentall procedure; (5) combined non-CABG procedures, comprising multivalve surgery and combined valve or aortic procedures without CABG; and (6) atrial septal defect repair. Isolated tricuspid valve surgery was grouped with mitral valve surgery because only two isolated tricuspid procedures were identified. Coronary ostial reimplantation performed as part of a Bentall procedure was not classified as CABG unless a coronary bypass graft was also performed.

For the expanded multivariable regression analyses, procedure type was consolidated into three clinically interpretable categories to reduce sparse outcome cells and improve model stability: CABG-containing procedures; isolated valve surgery, comprising aortic, mitral, or tricuspid valve procedures; and other non-CABG procedures, comprising combined non-CABG procedures, ascending aortic surgery, and atrial septal defect repair.

### 2.3. PBM Referral Pathway and Preoperative Anemia Screening

All patients scheduled for elective cardiac surgery underwent Hb screening during the preanesthetic assessment. Patients with Hb < 14.5 g/dL were referred to the PBM clinic for standardized etiological evaluation and, when clinically indicated, preoperative optimization.

The 14.5 g/dL threshold was adopted pragmatically by the local multidisciplinary team as a deliberately sensitive referral criterion. Its selection was informed by accumulated, although not formally analyzed, institutional clinical experience suggesting that patients entering cardiac procedures with Hb concentrations around 13 g/dL could have limited erythrocyte reserve in the context of anticipated surgical blood loss and cardiopulmonary bypass-related hemodilution. The higher referral threshold was therefore intended to allow early etiological assessment and, when clinically appropriate, the provision of additional erythrocyte reserve before surgery. It was not derived from a clinical trial or guideline establishing 14.5 g/dL as the optimal preoperative Hb concentration.

The same referral threshold was applied to women and men. Referral with Hb < 14.5 g/dL initiated further assessment but did not define anemia or constitute an automatic indication for pharmacological treatment. For the study analyses, preoperative anemia was defined as Hb < 13 g/dL, irrespective of sex, in accordance with contemporary perioperative PBM consensus recommendations and cardiac-surgery guidance [1,3,10,11,12]. Treatment decisions were individualized according to the identified abnormalities and clinical context, as described in Section 2.4 and Section 2.5.

### 2.4. Laboratory Assessment and Etiological Classification

Patients referred to the PBM clinic underwent a standardized laboratory assessment comprising a complete blood count; serum ferritin, transferrin saturation (TSAT), and reticulocyte hemoglobin content (CHr) for the assessment of iron status; vitamin B12 and folate; serum creatinine and estimated glomerular filtration rate (eGFR); and C-reactive protein (CRP), in accordance with contemporary cardiac-surgery and perioperative recommendations [1,3,6,10].

For this study, biochemical iron deficiency was considered present when at least one of the following criteria was met: serum ferritin < 30 ng/mL, TSAT < 20%, or CHr below the institutional cutoff of 29.1 pg. These markers were interpreted in conjunction with CRP and the clinical context because ferritin concentrations may be normal or elevated in the presence of inflammation, whereas CHr provides a more immediate measure of iron availability for erythropoiesis [3,6,10,20]. Iron-deficiency anemia was defined as biochemical iron deficiency with Hb < 13 g/dL, whereas non-anemic iron deficiency was defined as biochemical iron deficiency with Hb ≥ 13 g/dL.

Anemia with inflammation was defined as Hb < 13 g/dL with CRP > 10 mg/L. This category was further classified as mixed iron-deficiency and inflammatory anemia when biochemical iron deficiency was also present, or as probable anemia of chronic disease (ACD) when biochemical evidence of iron deficiency was absent. Vitamin B12 deficiency was defined as a serum vitamin B12 concentration < 200 pg/mL, and folate deficiency as a serum folate concentration < 2 ng/mL.

Severe renal impairment was defined as eGFR < 30 mL/min/1.73 m^2^ [21]. Preoperative anemia with severe renal impairment was defined descriptively as the coexistence of Hb < 13 g/dL and eGFR < 30 mL/min/1.73 m^2^. This category identified severe renal dysfunction as a potential contributor to anemia but did not establish it as the sole or confirmed cause. Coexisting biochemical iron deficiency, inflammation, vitamin B12 deficiency, and folate deficiency were recorded separately in their respective categories.

Anemia phenotypes and hematinic or renal abnormalities were summarized descriptively. Because individual patients could have more than one abnormality, these categories were not mutually exclusive.

### 2.5. Preoperative Treatment Algorithm

Treatment decisions followed a predefined institutional algorithm and were individualized according to baseline Hb concentration, iron status, inflammatory profile, vitamin status, renal function, body weight, contraindications, anticipated Hb response, procedural risk, and the time available before surgery.

Ferric carboxymaltose (FCM) was administered when clinically indicated and was withheld in patients with contraindications or known or suspected iron overload, including hemochromatosis. The FCM dose was determined according to baseline Hb concentration and body weight. Erythropoiesis-stimulating agents (ESAs) were used selectively, particularly in patients with probable ACD, mixed iron-deficiency and inflammatory anemia, or when the anticipated Hb response to iron alone was considered insufficient. The ESA dose was 450 international units (IU)/kg, with repeat dosing considered after reassessment of the Hb response. Vitamin B12 and folic acid supplementation was provided when the corresponding deficiencies were identified.

**Figure 1:**
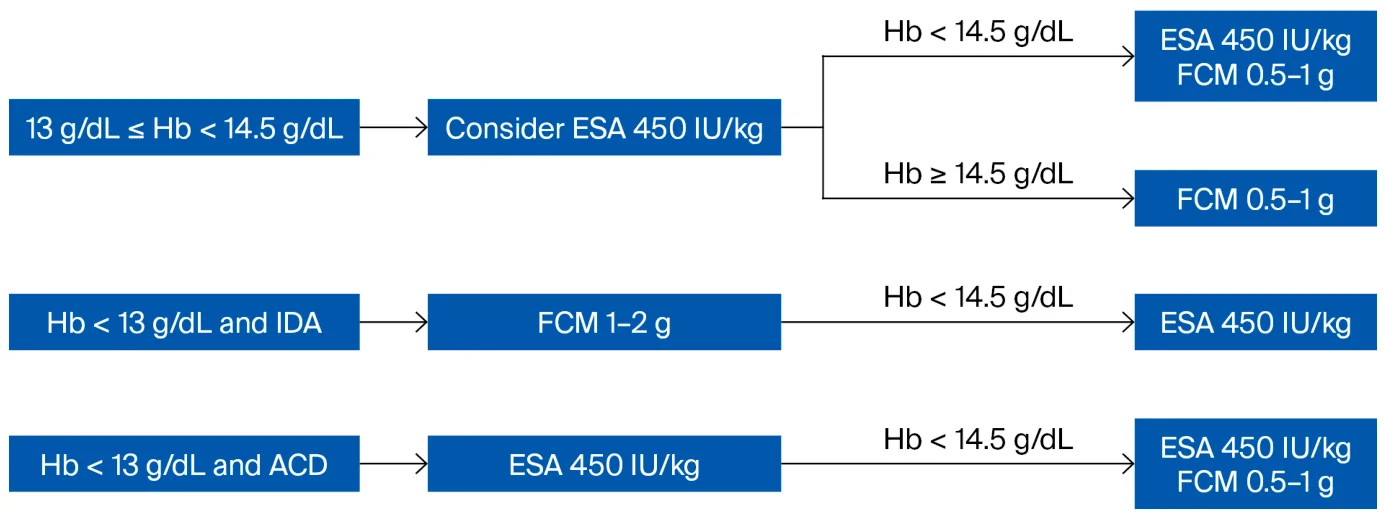
Institutional preoperative anemia-management algorithm within the PBM pathway. Treatment decisions were individualized and were not based solely on Hb concentration. FCM dosing was determined according to body weight and Hb concentration, whereas ESA use and repeat dosing were guided by the anticipated and observed Hb response. ACD, anemia of chronic disease; ESA, erythropoiesis-stimulating agent; FCM, ferric carboxymaltose; Hb, hemoglobin; IDA, iron-deficiency anemia; IU, international units; PBM, Patient Blood Management.

The institutional algorithm comprised three principal pathways. For patients with Hb concentrations of 13.0 to <14.5 g/dL, an ESA dose of 450 IU/kg and/or FCM 0.5–1 g could be considered according to iron status, anticipated Hb response, procedural risk, and the time available before surgery. For patients with Hb < 13 g/dL and iron-deficiency anemia, the protocol recommended a total FCM dose of 1–2 g; an additional ESA dose could be considered after reassessment if Hb remained <14.5 g/dL and further optimization was judged clinically appropriate. For patients with Hb < 13 g/dL and probable ACD, the protocol recommended an ESA dose of 450 IU/kg; repeat ESA dosing and/or FCM 0.5–1 g could be considered after reassessment if Hb remained <14.5 g/dL and further treatment was judged clinically appropriate. The treatment algorithm is summarized in Figure 1.

The exposure of interest was management through the structured institutional PBM pathway rather than receipt of any single pharmacological treatment. Depending on the identified hematological phenotype and clinical context, patients could receive FCM, ESA, vitamin B12 supplementation, folic acid supplementation, combinations of these treatments, or no pharmacological intervention. The study was therefore not designed to estimate the independent efficacy or causal contribution of any individual pharmacological or organizational component of the pathway.

### 2.6. Control Group Management and Secular Changes in Perioperative Practice

The historical No-PBM cohort comprised patients treated in 2017 and 2018 who retrospectively met the same PBM referral and study eligibility criteria applied to the PBM cohort. The same requirement for ascertainment of the principal comparative outcomes was applied to both cohorts; however, documentation and data-capture processes differed between study periods. During 2017–2018, preoperative assessment and perioperative care were delivered through routine clinical practice across anesthesiology, cardiac surgery, and other relevant clinical services. Patients were not evaluated within a dedicated multidisciplinary PBM clinic, and there was no standardized etiological-assessment pathway, protocolized preoperative optimization, or structured PBM documentation. Data for the historical cohort were therefore assembled retrospectively from multiple routine clinical sources. By contrast, the PBM pathway incorporated a dedicated multidisciplinary team, standardized etiological assessment, protocol-guided preoperative optimization, and structured clinical documentation. No protocolized hematology-supported pathway for preoperative optimization in cardiac surgery was available during the historical-control period, and no patient in the No-PBM cohort received preoperative FCM or ESA therapy.

Perioperative RBC transfusion decisions in both cohorts were made by the treating clinical teams and were individualized according to Hb concentration, ongoing bleeding, hemodynamic status, comorbidities, and clinical tolerance of anemia. Institutional guidance specified a reference Hb threshold of 9 g/dL during 2017–2019. This threshold was formally reduced to 8 g/dL in 2020 and was therefore the formal reference for the 2022 and 2023 PBM cohorts. Implementation of the revised guidance was gradual and varied across clinical teams. Throughout the study periods, the reference threshold was interpreted within the overall clinical context and did not constitute an automatic indication for transfusion. A further reduction to 7.5 g/dL was introduced in 2024, after the final index operation included in this study, and therefore did not affect the index-hospitalization transfusion outcomes. Apart from these threshold changes, no other formal changes in institutional RBC transfusion policy were documented during the operative years included in the analysis.

Other perioperative practices also evolved over time. Tranexamic acid was introduced into perioperative practice in 2020, although uptake was gradual and heterogeneous across clinical teams. Rotational thromboelastometry (ROTEM) had been available since 2017, but its use was initially inconsistent and became routinely integrated into clinical practice only in 2023. No formal enhanced-recovery pathway was implemented during the study period, and no formal changes were documented in cell-salvage practice, the composition of the cardiac surgical team, or intensive care unit and hospital discharge criteria. Potential secular effects were explored through the temporal sensitivity analyses described in Section 2.9.

### 2.7. Clinical, Safety, and Treatment-Response Outcomes

The primary clinical outcome was any allogeneic RBC transfusion, defined as receipt of at least one RBC unit during the index hospitalization for cardiac surgery.

Secondary transfusion outcomes included the total number of RBC units transfused and three mutually exclusive transfusion-intensity categories: no RBC transfusion, transfusion of 1–2 RBC units, and transfusion of >2 RBC units. Transfusion appropriateness was not independently adjudicated against prespecified criteria. These outcomes were therefore interpreted solely as measures of RBC exposure and transfusion intensity and did not distinguish appropriate from inappropriate transfusions.

Other secondary clinical outcomes included length of stay (LOS) in the thoracic surgery intensive care unit (T-SICU) and thoracic surgery unit (TSU), total postoperative hospital LOS, hospital readmission, total LOS accumulated during readmissions within 12 months, and all-cause mortality. Direct hospital costs were evaluated separately as economic outcomes, as described in Section 2.8.

Readmissions were assessed for 12 months after discharge from the index hospitalization and classified as occurring within 30 days, from >30 to ≤90 days, or from >90 days to 12 months. Cumulative readmission incidence was defined as the proportion of patients with at least one documented readmission within 30 days, 90 days, or 12 months after discharge. All-cause mortality was assessed cumulatively within 30 days, 90 days, and 12 months after surgery.

Exploratory postoperative safety outcomes available in both cohorts included myocardial infarction, stroke, documented thromboembolic events, infection, re-exploration for bleeding or cardiac tamponade, and decompensated heart failure. Events were identified retrospectively from the available medical records and classified as occurring within 30 days after surgery or from >30 days to 12 months. Cumulative 12-month incidence was defined as the occurrence of at least one documented event during either interval. These events were not independently adjudicated.

Among PBM patients, the same postoperative safety outcomes were also summarized descriptively according to FCM and ESA exposure. Pharmacological exposure was summarized as the number and proportion of patients receiving FCM, ESA, vitamin B12 supplementation, or folic acid supplementation; treatment categories were not mutually exclusive. FCM exposure was additionally summarized as the mean number of 500-mg vials administered per FCM-treated patient, whereas ESA exposure was summarized as the mean cumulative dose per ESA-treated patient, expressed in international units. Because treatment selection was clinically determined and exposure groups could overlap, no comparative or causal treatment-specific safety inference was intended.

Assessment of treatment-specific adverse events was limited by the absence of routine serum phosphate monitoring after FCM administration and by the lack of systematically collected data on acute infusion or hypersensitivity reactions, extravasation, treatment-emergent hypertension, treatment discontinuation, and other adverse effects specifically attributed to FCM or ESA. These outcomes therefore could not be assessed reliably.

Hematological response was assessed in treated PBM patients, defined in the dedicated Hb-response dataset by the presence of both pretreatment and post-treatment Hb measurements. The three prespecified assessment points were before treatment, at the first post-treatment reassessment, and at the final preoperative or day-of-surgery assessment. Patients who did not receive pharmacological treatment had no applicable post-treatment assessment and were excluded from the three-time-point longitudinal analysis. The intervals from pretreatment assessment to the first post-treatment reassessment and from post-treatment reassessment to surgery were calculated in weeks.

### 2.8. Cost Analysis

The economic component was conducted as a retrospective cost-consequence analysis from the hospital perspective. Clinical outcomes and the selected direct hospital cost components were reported separately rather than combined into a single measure of economic benefit. Health-related quality of life and other patient-reported outcomes were not incorporated into the analysis, quality-adjusted life-years were not estimated, and no incremental cost-effectiveness ratio or societal-perspective analysis was undertaken. Accordingly, the economic component was not intended to constitute a formal cost-effectiveness or cost-utility evaluation.

The analytical time horizon extended from the preoperative assessment to 12 months after discharge from the index hospitalization for cardiac surgery. Patient-level resource use comprised FCM, ESA therapy, RBC units, and T-SICU and TSU days during the index hospitalization and during readmissions occurring within 12 months. Only direct hospital costs captured within these predefined resource categories were included.

Only the predefined direct hospital resource categories were included. Costs outside the hospital perspective—including productivity losses, patient and caregiver time, informal caregiving, travel expenses, and other out-of-pocket costs—were excluded. Outpatient and community-care costs not captured by the institutional accounting system, rehabilitation and long-term-care costs, and costs beyond the 12-month follow-up period were also excluded. The analysis did not separately itemize the costs of the preoperative PBM assessment, diagnostic investigations, medications other than FCM and ESA, non-RBC blood products, personnel time, operating-room resources, procedures, or individual complications. Hospitalization costs were estimated using T-SICU and TSU daily costs rather than comprehensive patient-level microcosting; consequently, variation in resource intensity within an individual hospital day was not captured.

Unit costs were obtained from institutional hospital accounting data for 2024. Resource quantities observed in all study years were valued using the same 2024 institutional unit-cost schedule, and all costs were therefore expressed in 2024 euros. Historical nominal expenditures were not used, and no separate inflation adjustment was applied. This common-price-year approach prevented differences in nominal prices between calendar years from influencing the between-cohort comparison. Given the short preoperative interval and follow-up limited to 12 months after discharge, costs were not discounted.

The following unit costs were applied: FCM, EUR 174.40 per 1 g; ESA, EUR 30.52 per 30,000 IU and EUR 50.88 per 40,000 IU; RBC transfusion, EUR 104.20 per unit; T-SICU stay, EUR 2834 per day; and TSU stay, EUR 1267 per day.

For the purposes of this analysis, treatment-related cost was defined as the sum of FCM, ESA, and RBC transfusion costs. Index-hospitalization cost was calculated by multiplying the number of T-SICU and TSU days during the index admission by the corresponding institutional daily costs. Readmission cost was calculated similarly using the T-SICU and TSU days accumulated during readmissions within 12 months. Total hospitalization cost was defined as the sum of index-hospitalization and readmission costs. The study-defined overall direct hospital cost was calculated as the sum of treatment-related and total hospitalization costs. Mean cost differences were calculated as PBM minus No-PBM, with negative values indicating lower mean costs in the PBM cohort.

Patient-level costs of vitamin B12 and folic acid supplementation were not separately identifiable in the institutional accounting data and were therefore excluded from the base-case analysis.

### 2.9. Statistical Analysis

#### 2.9.1. Descriptive, Unadjusted, and Conventional Multivariable Analyses

Categorical variables were summarized as counts and percentages, and continuous variables as means and standard deviations. Where appropriate, estimates were accompanied by 95% confidence intervals (CIs). Confidence intervals for proportions were calculated using the Wilson method.

Unadjusted between-cohort comparisons were performed in the complete study population. Categorical variables were compared using the chi-square test or Fisher’s exact test, as appropriate. Non-cost continuous variables and outcomes were compared using independent-samples Student’s *t*-tests or Welch’s *t*-tests when the assumption of equal variances was not met. Longitudinal Hb and economic outcomes were analyzed separately, as described below.

Three separate multivariable binary logistic regression models were fitted for the following transfusion outcomes: any RBC transfusion, transfusion of 1–2 RBC units, and transfusion of >2 RBC units. The No-PBM cohort was the reference group. The primary individual-covariate model included management through the PBM pathway, age, sex, baseline Hb concentration, procedure type, New York Heart Association (NYHA) functional class, American Society of Anesthesiologists (ASA) Physical Status category, eGFR category, previous cardiac surgery, diabetes mellitus, preoperative anticoagulant use, and antiplatelet therapy.

For these models, procedure type was represented by three categories: CABG-containing procedures; isolated valve surgery, comprising aortic, mitral, or tricuspid valve procedures; and other non-CABG procedures, comprising combined non-CABG procedures, ascending aortic surgery, and atrial septal defect repair. Renal function was categorized as eGFR <30, 30–60, or >60 mL/min/1.73 m^2^. Serum creatinine was not entered concurrently with eGFR to avoid redundant adjustment. Results were reported as crude odds ratios (ORs) and adjusted odds ratios (aORs), with corresponding 95% CIs.

Continuous European System for Cardiac Operative Risk Evaluation II (EuroSCORE II) was not included in the primary individual-covariate model because it incorporates or overlaps with several of the clinical risk variables entered separately. A more parsimonious EuroSCORE-based sensitivity model was therefore fitted for each transfusion outcome. This model included management through the PBM pathway, continuous EuroSCORE II, baseline Hb concentration, procedure type, ASA Physical Status category, preoperative anticoagulant use, and antiplatelet therapy.

All between-cohort models evaluated management through the bundled institutional PBM pathway rather than exposure to individual pharmacological components. No comparative models were fitted separately for FCM, ESA, vitamin B12 supplementation, or folic acid supplementation because treatment selection was clinically determined, exposures could overlap, and component-specific comparisons would have been highly susceptible to confounding by indication.

Adjustment variables were restricted to factors measured before PBM-pathway management that were available in both cohorts and could be consistently harmonized across all study years. Patient-level left ventricular ejection fraction was not systematically available and could not be included. NYHA functional class was retained as an available measure of functional limitation but was not considered a substitute for direct assessment of left or right ventricular function.

Consistently harmonized patient-level data were also unavailable for several potentially relevant baseline characteristics, including chronic pulmonary disease, peripheral or cerebrovascular disease, pulmonary hypertension, frailty, body mass index, liver disease, platelet count, coagulation-test results, and the timing of preoperative anticoagulant or antiplatelet discontinuation. Some of these risk domains may have been partially captured by ASA Physical Status or EuroSCORE II, but they could not be evaluated individually.

Iron-deficiency status, inflammatory markers, vitamin B_12_, folate, and CHr were not included as common adjustment variables because they had not been systematically assessed in the historical No-PBM cohort. Detailed intraoperative variables—including cardiopulmonary bypass duration, aortic cross-clamp time, operative duration, the number of coronary grafts or valves treated, quantified blood loss, nadir hematocrit, and patient-level use of tranexamic acid, cell salvage, or ROTEM-guided management—were also not consistently available. Moreover, these factors were measured after assignment to PBM-pathway management and were therefore not entered into the primary pre-exposure models. Detailed procedure category and previous cardiac surgery were used as available preoperative proxies for operative complexity. No imputation was required for the covariates included in the final conventional models, and all 1374 patients were included.

Regression-model stability and diagnostic adequacy were assessed for each multivariable logistic regression model. The number of outcome events, predictor variables, and estimated predictor parameters, excluding the intercept, was recorded, and event-to-variable and event-to-parameter ratios were calculated.

Model convergence and the presence of finite coefficient estimates and standard errors were examined. Complete and quasi-complete separation were assessed using a linear-programming feasibility diagnostic, together with examination of convergence behavior, coefficient magnitude, standard errors, outcome patterns across categorical predictors, and the distribution of fitted probabilities. Multicollinearity was evaluated using variance inflation factors derived from the model design matrix.

Because transfusion of >2 RBC units had the lowest event-to-parameter ratio, additional analyses assessed the stability of this model. The influence of individual observations on the estimated PBM effect was examined using leave-one-out analysis: each patient was removed in turn, the full model was refitted, and the resulting PBM coefficient and OR were recorded. A Firth bias-reduced penalized logistic regression model was also fitted to evaluate potential small-sample and sparse-event bias. The Firth estimate was reported as an OR with a profile penalized-likelihood 95% CI.

Internal performance of the model for transfusion of >2 RBC units was additionally assessed using calibration-in-the-large, the calibration slope, the Brier score, and the area under the receiver operating characteristic curve (AUC). A calibration plot was constructed, and optimism-corrected estimates were obtained from 1000 bootstrap resamples. These analyses were used solely to assess internal model performance and potential overfitting and were not intended to develop or validate a clinical prediction model.

Conventional multivariable analyses were limited to transfusion outcomes because only these outcomes had sufficient events for the prespecified covariate set. Readmission at 30 days, 90 days, and 12 months was compared between the complete cohorts using unadjusted analyses. Twelve-month readmission was also assessed using propensity-score matching, inverse probability weighting, and adjacent-period matching. Expanded conventional models were not fitted for readmission because the number of events was insufficient relative to the required adjustment parameters.

Multivariable models were not fitted for mortality because the small number of events would have resulted in unstable estimates. Mortality was therefore compared between cohorts using Fisher’s exact test.

Available postoperative safety outcomes were summarized as counts and percentages. Events occurring within 30 days and from >30 days to 12 months were reported separately, together with cumulative 12-month incidence. Because most events were infrequent, between-cohort comparisons were performed using two-sided Fisher’s exact tests. These analyses were exploratory; no adjustment for multiple comparisons was applied, and no multivariable safety models were fitted.

Safety events among FCM-treated and ESA-treated PBM patients were summarized descriptively without formal comparisons between the treatment-exposure groups. The groups were not mutually exclusive, treatment allocation was clinically determined, and the recorded postoperative events had not been independently adjudicated as treatment-related.

#### 2.9.2. Longitudinal Hemoglobin-Response Analysis

The longitudinal Hb-response analysis included treated PBM patients with complete measurements at all three prespecified time points. No post-treatment Hb values were imputed.

Hb concentration at each assessment was summarized as the mean with standard deviation and 95% CI and as the median with interquartile range. Within-patient changes were evaluated using paired-samples *t*-tests for three prespecified comparisons: post-treatment vs. pretreatment Hb, final preoperative vs. post-treatment Hb, and final preoperative vs. pretreatment Hb. Results were reported as mean within-patient differences with 95% CIs. The three pairwise *p*-values were adjusted using the Holm method.

The intervals from the pretreatment assessment to the first post-treatment reassessment and from the post-treatment reassessment to surgery were expressed in weeks and summarized as medians with interquartile ranges.

The single preoperative Hb measurement available for the historical No-PBM cohort was used only as a descriptive cross-sectional comparator and was not included in the paired longitudinal analyses because the historical controls had no corresponding post-treatment measurements. Baseline Hb was compared between the complete PBM and No-PBM cohorts using the unadjusted methods described above.

#### 2.9.3. Economic Analyses

Cost distributions were inspected and were markedly right-skewed. Cost outcomes were summarized as arithmetic means with standard deviations and medians with interquartile ranges. Means were used for between-cohort comparisons because they represent the expected cost per patient and are relevant to hospital budget impact. The distributions of index-hospitalization cost and overall direct hospital cost were visualized by cohort using box-and-whisker plots on a logarithmic scale, with arithmetic means superimposed.

Uncertainty around mean cost differences was quantified using 1000 stratified nonparametric bootstrap resamples, with patients resampled separately within the PBM and No-PBM cohorts and percentile 95% CIs reported. For the unadjusted analyses, the mean cost difference was recalculated in each resample. Cost components with structural zero values—including readmission, RBC transfusion, FCM, and ESA costs—were summarized using means, standard deviations, medians, and interquartile ranges, and their unadjusted mean differences were estimated using the same bootstrap procedure.

For the strictly positive aggregate cost outcomes—index-hospitalization cost, total hospitalization cost, and overall direct hospital cost—covariate-adjusted generalized linear models were fitted using a Gamma distribution with a log link and robust sandwich standard errors. The models included management through the PBM pathway as the exposure and adjusted for the same prespecified covariates used in the primary individual-covariate transfusion model.

Results were reported as adjusted cost ratios with robust 95% CIs. Adjusted marginal mean costs under PBM and No-PBM management were estimated using recycled predictions. Each patient was assigned in turn to each exposure condition while all other covariates were retained at their observed values, and the resulting predicted costs were averaged across the complete study population. To estimate adjusted marginal mean differences and their 95% CIs, the complete generalized linear model was refitted in each bootstrap resample before the marginal means were recalculated.

Economic sensitivity analyses included exclusion of readmission costs; one-way variation of each institutional unit cost by ±20%; combined conservative and favorable cost scenarios; use of an inverse-Gaussian generalized linear model with a log link in place of the Gamma model for overall direct hospital cost; and repetition of the overall direct-cost analysis in the propensity-score-matched, inverse-probability-weighted, and adjacent-period matched populations. In the conservative combined scenario, FCM and ESA unit costs were increased by 20%, whereas RBC, T-SICU, and TSU unit costs were reduced by 20%. The favorable scenario applied the inverse assumptions.

#### 2.9.4. Propensity-Score Analyses

Propensity-score matching (PSM) and stabilized inverse probability of treatment weighting (IPTW) were used as sensitivity analyses to reduce confounding from measured baseline differences. The propensity score, defined as the probability of management through the PBM pathway, was estimated using logistic regression including age, sex, the six-category surgical classification, baseline Hb concentration, NYHA functional class (I–II vs. III–IV), ASA Physical Status category (I–II vs. III–IV), eGFR category (<30, 30–60, or >60 mL/min/1.73 m^2^), previous cardiac surgery, diabetes mellitus, preoperative anticoagulant use, and antiplatelet therapy. Continuous EuroSCORE II was not included because of overlap with several model covariates but was evaluated as an additional balance diagnostic. Calendar year was not included because the PBM and No-PBM cohorts occupied non-overlapping periods; potential temporal effects were examined separately. No imputation was required, and propensity-score overlap was examined in both analyses.

For PSM, PBM patients were matched 1:1 to No-PBM controls without replacement using optimal matching, exact matching on the six-category surgical classification, and a caliper of 0.2 standard deviations of the logit of the propensity score. Patients without an eligible match were excluded. Covariate balance was assessed using standardized mean differences (SMDs), with an absolute SMD < 0.10 considered acceptable. Binary outcomes were analyzed using conditional logistic regression, and continuous outcomes using paired analyses. Skewed cost outcomes were analyzed using nonparametric bootstrap resampling of matched pairs. Results were reported as matched odds ratios or mean differences with 95% CIs.

Stabilized inverse probability of treatment weights targeting the average treatment effect were derived from the same propensity-score model. Weight distributions and effective sample size were examined, and the need for weight truncation was assessed from the observed distribution. Post-weighting balance was evaluated using the same SMD threshold. Binary outcomes were analyzed using weighted logistic regression and continuous clinical outcomes using weighted linear regression, both with robust sandwich standard errors. Results were reported as weighted odds ratios or mean differences with 95% CIs. Overall direct hospital cost in the IPTW population was compared using weighted mean estimates. The 95% CI for the weighted mean difference was obtained from 1000 stratified nonparametric bootstrap resamples, with the propensity-score model and stabilized weights re-estimated in each resample.

#### 2.9.5. Temporal Sensitivity Analyses

Potential secular trends were explored through two additional analyses. First, clinical and economic outcomes were compared between the two pre-implementation years, 2017 and 2018. Categorical and non-cost continuous outcomes were analyzed using the unadjusted methods described above, whereas mean economic differences were evaluated using stratified nonparametric bootstrap CIs. This analysis examined whether changes in transfusion exposure or intensity, postoperative length of stay, readmission, or direct hospital costs were already apparent before implementation of the structured PBM pathway.

Second, an adjacent-period risk-matched analysis compared No-PBM patients who underwent surgery in 2018 with PBM patients who underwent surgery in 2019. This comparison minimized the temporal separation between cohorts and preceded the documented changes in institutional transfusion guidance and tranexamic acid practice introduced in 2020.

For the adjacent-period analysis, the propensity score was re-estimated in the restricted 2018–2019 population using the same available baseline covariates as in the primary propensity-score model. Patients were matched 1:1 without replacement, with exact matching on detailed surgical category, NYHA category, and previous cardiac surgery. Mahalanobis distance within the propensity-score caliper was used to optimize balance across the remaining covariates.

Balance in the adjacent-period matched population was evaluated using SMDs, with an absolute value < 0.10 considered acceptable. Binary and non-cost continuous outcomes were analyzed using the same matched methods as in the primary PSM analysis. Overall direct hospital cost was evaluated using the paired mean difference and a 95% CI obtained through nonparametric bootstrap resampling of matched pairs.

All statistical tests were two-sided, and a *p*-value < 0.05 was considered statistically significant. Analyses were performed using IBM SPSS Statistics version 30.0 (IBM Corp., Armonk, NY, USA) and Python version 3.13.5 (Python Software Foundation, Wilmington, DE, USA). Given the retrospective historical-control design, all between-cohort estimates were interpreted as associations rather than causal effects.

## 3. Results

### 3.1. Study Population and Baseline Characteristics

**Figure 2:**
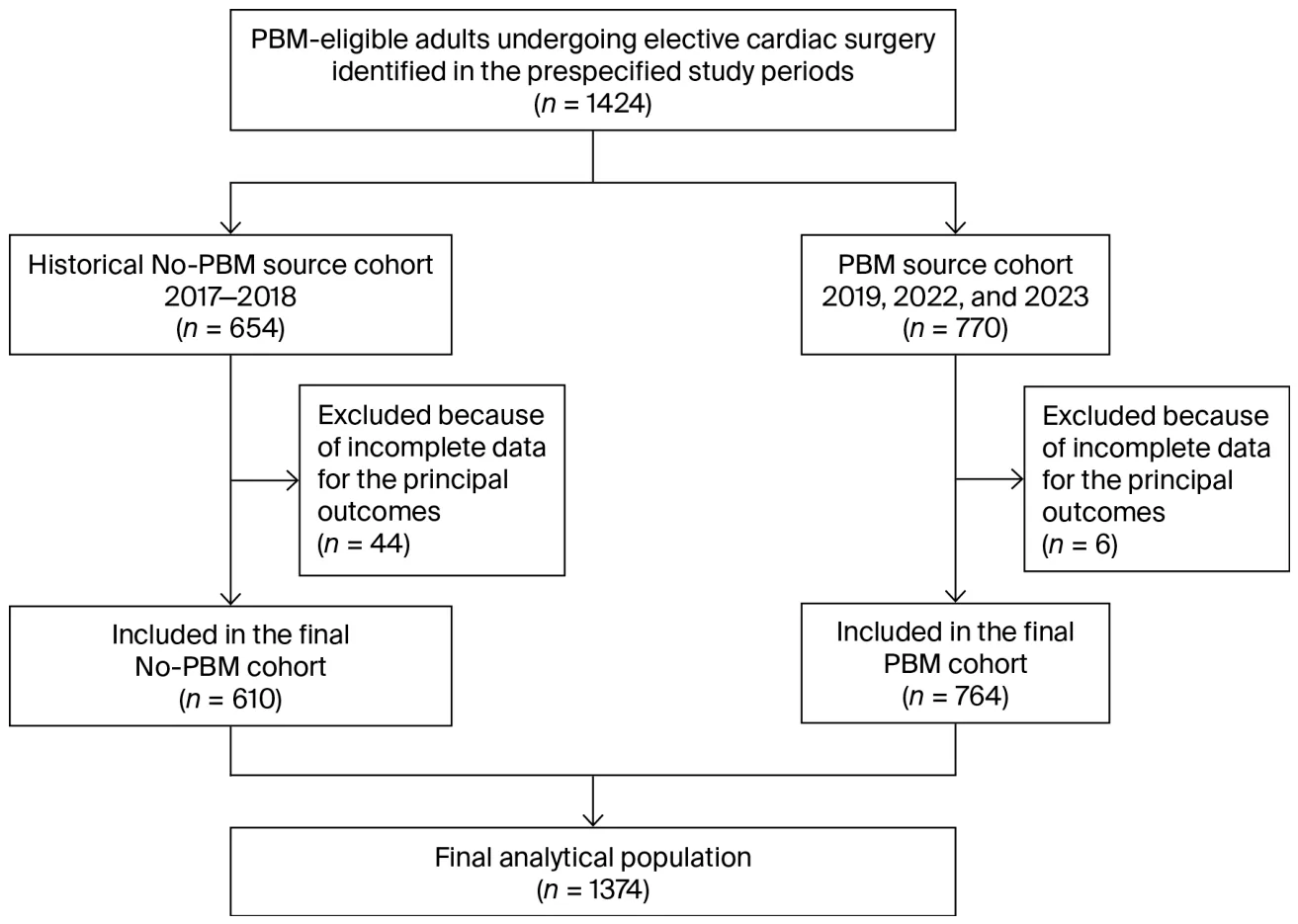
Participant flow through cohort assembly and final analytical inclusion. The historical No-PBM source cohort comprised PBM-eligible adults who underwent elective cardiac surgery in 2017 and 2018, before implementation of the structured Patient Blood Management pathway. The PBM source cohort comprised eligible patients managed through the pathway in 2019, 2022, and 2023. Of 1424 initially identified patients, 50 were excluded because of incomplete data for the principal outcomes: 44 from the No-PBM cohort and 6 from the PBM cohort. The final analytical population comprised 1374 patients, including 610 No-PBM controls and 764 PBM patients. Calendar years 2020 and 2021 were not included because the COVID-19 pandemic substantially disrupted elective cardiac-surgery activity. No-PBM, historical control cohort not managed through the structured Patient Blood Management pathway; PBM, Patient Blood Management.

A total of 1424 PBM-eligible adults who underwent elective cardiac surgery during the prespecified study periods were initially identified. The historical No-PBM source cohort comprised 654 patients treated in 2017–2018, of whom 44 were excluded because of incomplete data for the principal outcomes, leaving 610 patients in the final No-PBM cohort. The PBM source cohort comprised 770 patients managed through the institutional pathway in 2019, 2022, and 2023; six were excluded because of incomplete principal-outcome data, leaving 764 patients in the final PBM cohort. Calendar years 2020 and 2021 were not included because the coronavirus disease 2019 (COVID-19) pandemic substantially disrupted elective cardiac-surgery activity. The final analytical population therefore comprised 1374 patients: 764 PBM patients and 610 historical No-PBM controls (Figure 2). The proportion excluded because of incomplete principal-outcome data was higher in the historical No-PBM source cohort than in the PBM source cohort (44/654 [6.7%] vs. 6/770 [0.8%]), consistent with the less standardized and more heterogeneous clinical documentation available before implementation of the dedicated PBM pathway.

The distribution across the six detailed procedure categories did not differ significantly between cohorts (overall *p* = 0.415) (Table 1). The most frequent categories were aortic valve surgery (34.6% vs. 33.4%), CABG-containing procedures (29.2% vs. 32.1%), and combined non-CABG procedures (26.6% vs. 27.4%) in the PBM and No-PBM cohorts, respectively.

Several clinically relevant baseline characteristics differed between cohorts. NYHA class III–IV was more frequent in the PBM cohort (31.7% vs. 15.2%; *p* < 0.001), as was ASA class III–IV (98.0% vs. 92.6%; *p* < 0.001). Renal-function distribution also differed significantly (overall *p* < 0.001). An eGFR of 30–60 mL/min/1.73 m^2^ was more frequent in the PBM cohort (20.3% vs. 9.0%), whereas an eGFR > 60 mL/min/1.73 m^2^ was more frequent in the No-PBM cohort (88.4% vs. 77.5%). Severe renal impairment, defined as eGFR < 30 mL/min/1.73 m^2^, was similarly uncommon in both groups (2.2% vs. 2.6%). Overall, an eGFR ≤ 60 mL/min/1.73 m^2^ was present in 22.5% of PBM patients and 11.6% of No-PBM controls.

Previous cardiac surgery was more frequent in the PBM cohort (3.9% vs. 1.0%; *p* < 0.001), whereas diabetes mellitus was more frequent in the No-PBM cohort (32.5% vs. 25.5%; *p* = 0.005). Mean continuous EuroSCORE II was slightly higher in the PBM cohort (2.16 ± 2.80% vs. 1.93 ± 1.48%), although the difference was not statistically significant (*p* = 0.055). The corresponding absolute SMD was 0.101, indicating a small baseline imbalance. The proportion of patients with EuroSCORE II > 2% was similar between cohorts (30.5% vs. 28.0%; *p* = 0.319). These measured baseline differences were addressed in the multivariable and propensity-score analyses.

### 3.2. Preoperative Anemia Phenotype and Hematinic Abnormalities

Among the 764 patients in the PBM cohort, 212 (27.7%) met the study definition of preoperative anemia. Biochemical iron deficiency, irrespective of anemia status, was identified in 203 patients (26.6%); 101 (13.2% of the overall cohort) had iron-deficiency anemia and 102 (13.4%) had non-anemic iron deficiency.

Anemia with inflammation was identified in 28 patients (3.7%), including 20 (2.6%) with mixed iron-deficiency and inflammatory anemia and 8 (1.0%) with probable anemia of chronic disease. Preoperative anemia with severe renal impairment was present in 5 patients (0.7%). Folate deficiency was identified in 131 patients (17.1%), and vitamin B12 deficiency in 25 patients (3.3%). The distribution of preoperative anemia phenotypes and hematinic abnormalities is shown in Table 2.

### 3.3. PBM Treatment Exposure and Hemoglobin Response

Pharmacological treatment was administered to 442 of 764 PBM patients (57.9%). FCM was administered to 355 patients (46.5%), and ESA to 69 patients (9.0%). Sixty-six patients (8.6%) received both FCM and ESA, resulting in 358 patients (46.9%) who received FCM, ESA, or both. Folic acid supplementation was administered to 131 patients (17.1%), and vitamin B12 supplementation to 25 patients (3.3%). Treatment categories were not mutually exclusive because patients with multiple correctable abnormalities could receive more than one therapy.

Among FCM-treated patients, the mean number of 500-mg vials administered per patient was 2.02 (95% CI, 1.98–2.05). Among ESA-treated patients, the mean cumulative dose was 99,275 IU (95% CI, 86,934–111,616).

Complete sequential pretreatment, post-treatment, and final preoperative or day-of-surgery Hb measurements were available for 442 treated PBM patients. Mean Hb increased from 12.76 ± 1.20 g/dL before treatment (95% CI, 12.65–12.88) to 14.77 ± 0.56 g/dL at the first post-treatment reassessment (95% CI, 14.72–14.83). The mean within-patient increase was 2.01 g/dL (95% CI, 1.91–2.10; Holm-adjusted *p* < 0.001).

At the final preoperative or day-of-surgery assessment, mean Hb was 13.74 ± 1.05 g/dL (95% CI, 13.64–13.84). This concentration remained 0.97 g/dL above the pretreatment value (95% CI, 0.85–1.10; Holm-adjusted *p* < 0.001) but was 1.03 g/dL below the post-treatment value (mean difference, −1.03 g/dL; 95% CI, −1.12 to −0.95; Holm-adjusted *p* < 0.001).

The median interval from pretreatment assessment to the first post-treatment reassessment was 4.0 weeks (IQR, 3.0–5.0; *n* = 442). The median interval from post-treatment reassessment to surgery was 6.0 weeks (IQR, 4.0–8.2; *n* = 442).

**Figure 3:**
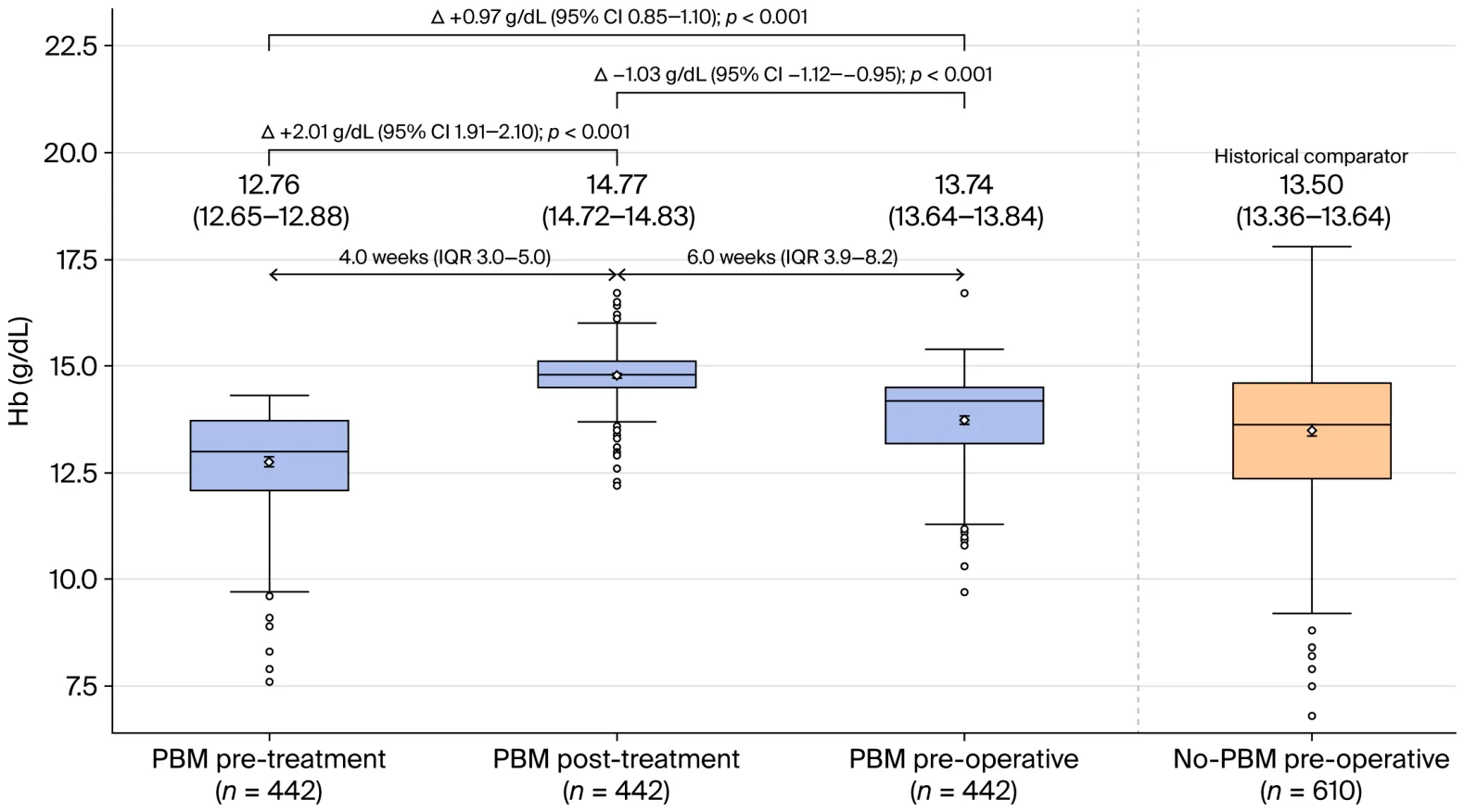
Longitudinal hemoglobin response among treated PBM patients, with the historical No-PBM cohort shown as a descriptive cross-sectional comparator. The first three boxplots represent the same 442 treated PBM patients with complete pretreatment, first post-treatment, and final preoperative or day-of-surgery Hb measurements. The No-PBM boxplot represents the preoperative Hb concentration of 610 historical controls and was not included in the paired longitudinal analyses. Boxes show medians and interquartile ranges; whiskers extend to 1.5 times the interquartile range, and circles indicate observations beyond the whiskers. White diamonds and error bars indicate arithmetic means and 95% confidence intervals. Brackets show mean within-patient differences with 95% confidence intervals. Pairwise comparisons were performed using paired-samples *t*-tests with Holm adjustment for three comparisons. Median intervals between assessment points are displayed above the corresponding segments. CI, confidence interval; Hb, hemoglobin; IQR, interquartile range; No-PBM, historical control cohort not managed through the structured Patient Blood Management pathway; PBM, Patient Blood Management.

For descriptive context, mean preoperative Hb in the 610 historical No-PBM controls was 13.50 ± 1.70 g/dL (95% CI, 13.36–13.64). This cross-sectional value is shown in Figure 3 but was not included in the paired longitudinal comparisons of treated PBM patients.

### 3.4. Red Blood Cell Transfusion Outcomes

In unadjusted comparisons, any allogeneic RBC transfusion during the index hospitalization was less frequent in the PBM cohort than in the No-PBM cohort, occurring in 132 of 764 patients (17.3%) and 239 of 610 patients (39.2%), respectively (*p* < 0.001). Correspondingly, 632 PBM patients (82.7%) and 371 No-PBM patients (60.8%) received no RBC transfusion.

Transfusion of 1–2 RBC units occurred in 112 PBM patients (14.7%) and 147 No-PBM patients (24.1%; *p* < 0.001). Transfusion of >2 RBC units was also less frequent in the PBM cohort, occurring in 20 patients (2.6%) compared with 92 No-PBM patients (15.1%; *p* < 0.001).

### 3.5. Length of Stay, Readmissions and Mortality

Postoperative LOS was shorter in the PBM cohort than in the No-PBM cohort. Mean T-SICU LOS was 2.88 days in the PBM cohort and 3.90 days in the No-PBM cohort (*p* < 0.001), whereas mean TSU LOS was 5.17 and 6.17 days, respectively (*p* = 0.002). Mean total postoperative LOS was therefore 8.05 days in the PBM cohort compared with 10.07 days in the No-PBM cohort (*p* < 0.001) (Table 3).

Among patients readmitted within 12 months, mean T-SICU LOS during readmission was shorter in the PBM cohort than in the No-PBM cohort (0.68 vs. 2.56 days; *p* = 0.006). Mean TSU LOS during readmission did not differ significantly between groups (14.60 vs. 11.09 days; *p* = 0.177), nor did mean total readmission LOS (15.28 vs. 13.64 days; *p* = 0.533).

Cumulative readmission rates were similar between cohorts at 30 and 90 days. Readmission within 30 days occurred in 35 of 764 PBM patients (4.6%) and 29 of 610 No-PBM patients (4.8%; *p* = 0.898). By 90 days, cumulative readmission had occurred in 38 PBM patients (5.0%) and 40 No-PBM patients (6.6%; *p* = 0.241). At 12 months, cumulative readmission was lower in the PBM cohort than in the No-PBM cohort (41/764 [5.4%] vs. 49/610 [8.0%]; *p* = 0.049).

All-cause mortality was low in both cohorts and did not differ significantly at any assessed time point. Thirty-day mortality occurred in 3 of 764 PBM patients (0.39%) and 2 of 610 No-PBM patients (0.33%; *p* = 1.000). Cumulative 90-day mortality was 5 of 764 (0.65%) and 3 of 610 (0.49%), respectively (*p* = 1.000). At 12 months, cumulative mortality was 5 of 764 PBM patients (0.65%) and 4 of 610 No-PBM patients (0.66%; *p* = 1.000).

### 3.6. Direct Hospital Costs

Direct hospital cost distributions were markedly right-skewed. Costs are therefore presented as arithmetic means with standard deviations and medians with interquartile ranges; 95% confidence intervals for mean differences were estimated using stratified nonparametric bootstrap resampling (Table 4).

**Figure 4:**
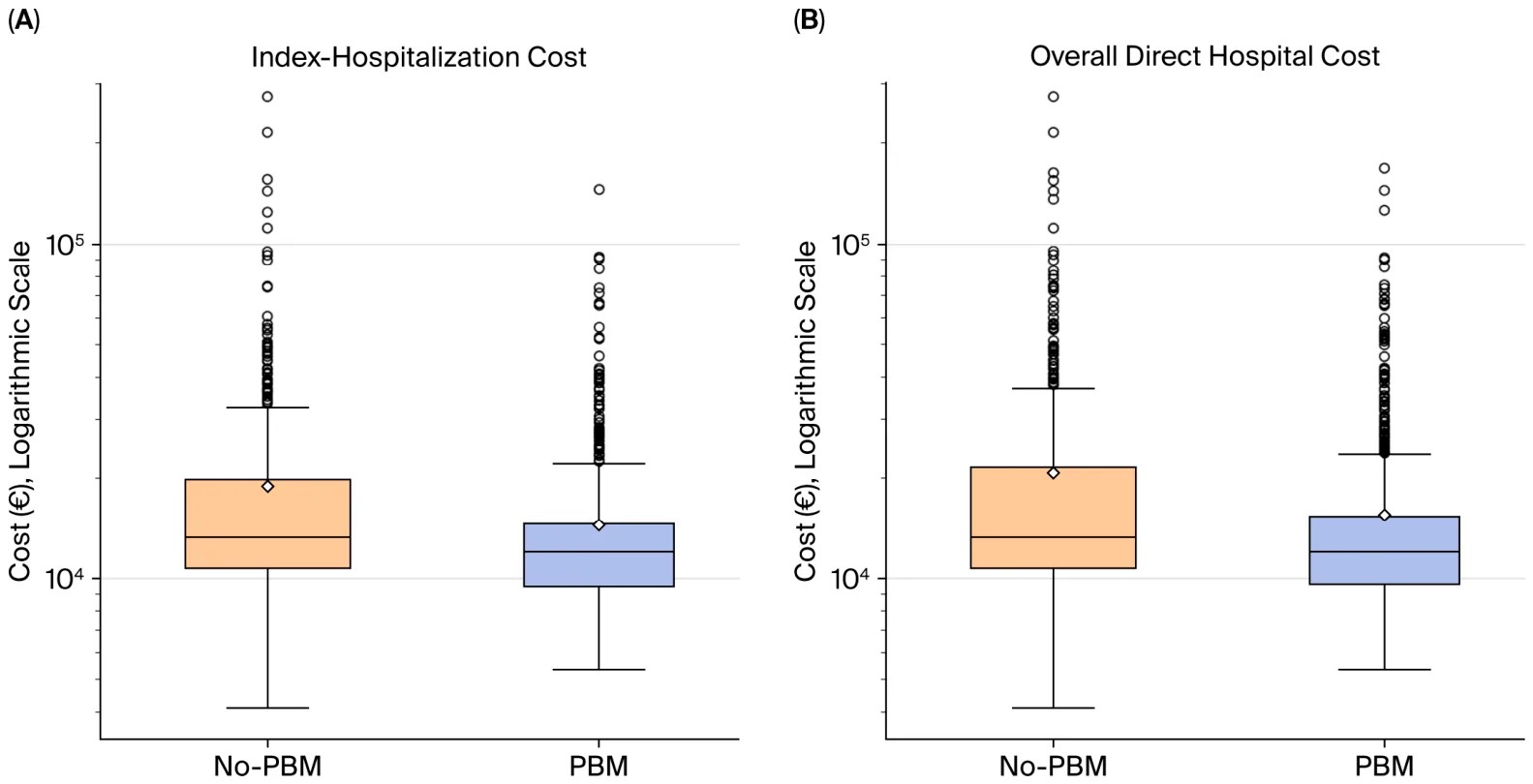
Distribution of direct hospital costs by study cohort. Panel (**A**) shows index-hospitalization cost, and Panel (**B**) shows overall direct hospital cost. Boxes show medians and interquartile ranges; whiskers extend to 1.5 times the interquartile range, and circles indicate observations beyond the whiskers. Diamonds indicate arithmetic means. The vertical axis is shown on a logarithmic scale because cost distributions were markedly right-skewed. All costs are expressed in 2024 euros. Index-hospitalization cost comprised T-SICU and TSU costs during the index admission. Overall direct hospital cost comprised treatment-related cost, index-hospitalization cost, and readmission cost within 12 months. No-PBM, historical control cohort not managed through the structured Patient Blood Management pathway; PBM, Patient Blood Management; T-SICU, thoracic surgery intensive care unit; TSU, thoracic surgery unit.

Mean overall direct hospital cost was EUR 15,632.60 per patient in the PBM cohort and EUR 20,588.38 in the No-PBM cohort. The corresponding medians were EUR 12,003 and EUR 13,870, respectively. The unadjusted mean difference was −EUR 4955.78 per patient (bootstrap 95% CI, −EUR 7008.91 to −EUR 3039.86). The distributions of index-hospitalization and overall direct hospital costs are shown in Figure 4.

Mean FCM cost was EUR 81.72 per PBM patient, compared with EUR 0 in the historical No-PBM cohort, and mean ESA cost was EUR 11.05 per PBM patient. Mean RBC transfusion cost was lower in the PBM cohort (EUR 30.82 vs. EUR 114.79), corresponding to a mean difference of −EUR 83.97 (bootstrap 95% CI, −EUR 105.25 to −EUR 64.98). Overall, mean treatment-related cost was EUR 123.60 in the PBM cohort and EUR 114.79 in the No-PBM cohort; the mean difference was EUR 8.81 (bootstrap 95% CI, −EUR 11.60 to EUR 28.98).

Mean index-hospitalization cost was EUR 14,419.06 in the PBM cohort and EUR 18,863.40 in the No-PBM cohort, corresponding to a mean difference of −EUR 4444.34 (bootstrap 95% CI, −EUR 6339.47 to −EUR 2670.26). Mean readmission cost was EUR 1089.94 and EUR 1610.19, respectively, with a mean difference of −EUR 520.25 (bootstrap 95% CI, −EUR 1258.54 to EUR 186.66). Mean total hospitalization cost was EUR 15,509.00 in the PBM cohort and EUR 20,473.59 in the No-PBM cohort (mean difference, −EUR 4964.59; bootstrap 95% CI, −EUR 6988.04 to −EUR 3120.12).

In the covariate-adjusted Gamma generalized linear model, management through the PBM pathway was associated with lower mean overall direct hospital cost (adjusted cost ratio, 0.75; robust 95% CI, 0.68–0.82; *p* < 0.001). Adjusted marginal mean costs were EUR 15,544 under PBM and EUR 20,739 under No-PBM management, corresponding to an adjusted marginal mean difference of −EUR 5196 (bootstrap 95% CI, −EUR 6965 to −EUR 3485).

Adjusted results for index-hospitalization and total hospitalization costs were concordant, with cost ratios of 0.76 (95% CI, 0.69–0.83) and 0.75 (95% CI, 0.68–0.82), respectively. The inverse-Gaussian sensitivity model yielded a nearly identical overall direct-cost ratio of 0.75 (95% CI, 0.68–0.82).

Economic sensitivity analyses were directionally consistent with the base-case findings. Excluding readmission costs yielded a mean difference of −EUR 4436 per patient (bootstrap 95% CI, −EUR 6182 to −EUR 2792). Under the combined deterministic ±20% unit-cost scenarios, the estimated mean between-cohort difference ranged from −EUR 3928 to −EUR 5984 per patient. Cost differences also remained negative in the propensity-score-matched, inverse-probability-weighted, and adjacent-period matched analyses. Complete economic sensitivity analyses are presented in Supplementary Table S7.

### 3.7. Expanded Multivariable Analyses and Regression Diagnostics

The expanded multivariable logistic-regression analyses included all 1374 patients because no data were missing for the covariates entered in the models. In the primary individual-covariate model, management through the PBM pathway remained associated with lower odds of any RBC transfusion after adjustment for age, sex, baseline Hb concentration, procedure type, NYHA functional class, ASA Physical Status category, eGFR category, previous cardiac surgery, diabetes mellitus, preoperative anticoagulant use, and antiplatelet therapy (aOR, 0.30; 95% CI, 0.23–0.39; *p* < 0.001).

Management through the PBM pathway was also associated with lower adjusted odds of transfusion of 1–2 RBC units (aOR, 0.49; 95% CI, 0.37–0.66; *p* < 0.001) and >2 RBC units (aOR, 0.14; 95% CI, 0.08–0.24; *p* < 0.001). Results were concordant in the EuroSCORE-based sensitivity models, with adjusted ORs of 0.32 (95% CI, 0.25–0.42), 0.53 (95% CI, 0.40–0.70), and 0.16 (95% CI, 0.09–0.26), respectively (all *p* < 0.001) (Table 5).

The numbers of outcome events were 371 for any RBC transfusion, 259 for transfusion of 1–2 RBC units, and 112 for transfusion of >2 RBC units. The primary model included 12 predictor variables represented by 14 predictor parameters, excluding the intercept. This corresponded to 30.9, 21.6, and 9.3 events per predictor variable, respectively, and 26.5, 18.5, and 8.0 events per predictor parameter.

All three maximum-likelihood models converged and yielded finite coefficient estimates. For the >2-unit transfusion model, predicted probabilities ranged from 0.003 to 0.307, and multicollinearity was low, with a maximum variance inflation factor of 1.57. Leave-one-out refitting yielded PBM OR estimates ranging from 0.134 to 0.147, indicating that the association was not dependent on any single observation.

The Firth bias-reduced sensitivity analysis yielded an estimate closely concordant with the conventional maximum-likelihood model (OR, 0.15; 95% profile penalized-likelihood CI, 0.09–0.24; *p* < 0.001). Together with model convergence and finite coefficients, this provided no evidence that the observed association was driven by complete separation.

For the >2-unit transfusion model, the apparent area under the receiver operating characteristic curve was 0.753, and the apparent Brier score was 0.0697. After optimism correction using 1000 bootstrap resamples, the AUC was 0.718 and the Brier score was 0.0722. Bootstrap-corrected calibration-in-the-large was 0.006, and the calibration slope was 0.720, indicating some model optimism consistent with the lower event-to-parameter ratio. The precise magnitude of the association should therefore be interpreted cautiously. Full regression coefficients and diagnostic results are presented in Supplementary Tables S5 and S6, and the calibration plot is shown in Supplementary Figure S3.

### 3.8. Propensity-Score and Temporal Sensitivity Analyses

The primary propensity-score-matching analysis yielded 519 matched pairs, comprising 519 PBM patients and 519 No-PBM controls. After matching, all covariates included in the propensity-score model achieved acceptable balance, with absolute standardized mean differences below 0.10 and a maximum absolute standardized mean difference of 0.059. Continuous EuroSCORE II, evaluated as an additional balance diagnostic, was also well balanced after matching (absolute standardized mean difference, 0.027). Detailed covariate-balance results are presented in Supplementary Table S1 and Figure S1.

In the matched population, any RBC transfusion occurred in 86 of 519 PBM patients (16.6%) and 208 of 519 No-PBM controls (40.1%), corresponding to a matched OR of 0.29 (95% CI, 0.21–0.39; *p* < 0.001). Transfusion of 1–2 RBC units occurred in 74 PBM patients (14.3%) and 127 No-PBM controls (24.5%; matched OR, 0.50; 95% CI, 0.36–0.69; *p* < 0.001), whereas transfusion of >2 RBC units occurred in 12 PBM patients (2.3%) and 81 No-PBM controls (15.6%; matched OR, 0.13; 95% CI, 0.07–0.24; *p* < 0.001). Post-weighting balance is also summarized in Supplementary Table S1 and Figure S1.

Mean RBC exposure was 0.28 units in the matched PBM group and 1.13 units in the matched No-PBM group, corresponding to a paired mean difference of −0.85 units (95% CI, −1.07 to −0.63; *p* < 0.001). Mean T-SICU LOS was 2.66 and 4.01 days, respectively (mean difference, −1.34 days; 95% CI, −1.91 to −0.77; *p* < 0.001), while mean TSU LOS was 5.07 and 6.24 days (mean difference, −1.17 days; 95% CI, −1.86 to −0.49; *p* < 0.001). Mean total postoperative LOS was therefore 7.74 days in the matched PBM group and 10.25 days in the matched No-PBM group, corresponding to a paired mean difference of −2.51 days (95% CI, −3.47 to −1.56; *p* < 0.001).

Twelve-month readmission occurred in 24 of 519 PBM patients (4.6%) and 43 of 519 No-PBM controls (8.3%), corresponding to a matched OR of 0.55 (95% CI, 0.33–0.91; *p* = 0.025). Mean overall direct hospital cost was EUR 14,850 in the matched PBM group and EUR 21,051 in the matched No-PBM group. The paired mean difference was −EUR 6201, with a paired-bootstrap 95% CI of −EUR 8481 to −EUR 4195. The complete matched and weighted outcome results are presented in Table 6.

The comparison of the two pre-implementation years did not identify evidence of a pre-existing improvement in the principal clinical or economic outcomes. Any RBC transfusion occurred in 114 of 312 patients (36.5%) in 2017 and 125 of 298 patients (41.9%) in 2018 (*p* = 0.171), whereas transfusion of >2 RBC units occurred in 13.8% and 16.4%, respectively (*p* = 0.359). Mean total postoperative LOS was 10.27 versus 9.86 days (*p* = 0.603), and 12-month readmission occurred in 7.4% versus 8.7% (*p* = 0.539). Mean overall direct hospital cost was EUR 20,445 in 2017 and EUR 20,739 in 2018, with a mean difference of EUR 294 (bootstrap 95% CI, −EUR 3057 to EUR 4035). Full results are presented in Supplementary Table S2.

The adjacent-period sensitivity analysis comparing 2018 No-PBM patients with 2019 PBM patients yielded 134 matched pairs. All covariates used in the matching procedure achieved acceptable balance, with absolute standardized mean differences below 0.10 and a maximum value of 0.063.

Any RBC transfusion occurred in 18 of 134 PBM patients (13.4%) and 54 of 134 matched No-PBM controls (40.3%), corresponding to a matched OR of 0.28 (95% CI, 0.15–0.51; *p* < 0.001). Transfusion of 1–2 RBC units occurred in 16 PBM patients (11.9%) and 31 No-PBM controls (23.1%; matched OR, 0.48; 95% CI, 0.26–0.91; *p* = 0.025), whereas transfusion of >2 RBC units occurred in 2 PBM patients (1.5%) and 23 No-PBM controls (17.2%; matched OR, 0.09; 95% CI, 0.02–0.37; *p* < 0.001).

Mean total postoperative LOS was 7.02 days in the 2019 PBM group and 9.21 days in the matched 2018 No-PBM group, corresponding to a paired mean difference of −2.19 days (95% CI, −3.96 to −0.41). Mean overall direct hospital cost was EUR 13,656 in the 2019 PBM group and EUR 19,544 in the matched 2018 No-PBM group. The paired mean difference was −EUR 5888, with a paired-bootstrap 95% CI of −EUR 10,707 to −EUR 1833.

Twelve-month readmission did not differ significantly between the adjacent-period groups, occurring in 6 of 134 PBM patients (4.5%) and 8 of 134 No-PBM controls (6.0%; matched OR, 0.75; 95% CI, 0.26–2.16; *p* = 0.59). The adjacent-period balance and outcome results are presented in Supplementary Tables S3 and S4 and Figure S2.

### 3.9. Available Postoperative Safety Outcomes

Recorded postoperative safety events were generally uncommon in both cohorts (Table 7).

Within 30 days after surgery, myocardial infarction occurred in no PBM patients and in 2 of 610 No-PBM controls (0% vs. 0.33%; *p* = 0.197). Stroke occurred in 4 of 764 PBM patients and 2 of 610 No-PBM controls (0.52% vs. 0.33%; *p* = 0.699), whereas documented thromboembolic events occurred in no PBM patients and in one No-PBM control (0% vs. 0.16%; *p* = 0.444). Postoperative infection within 30 days was documented in 25 PBM patients and 12 No-PBM controls (3.27% vs. 1.97%; *p* = 0.179).

At 12 months, cumulative myocardial infarction occurred in 1 of 764 PBM patients and 2 of 610 No-PBM controls (0.13% vs. 0.33%; *p* = 0.588). Cumulative stroke occurred in 5 patients in each cohort (0.65% vs. 0.82%; *p* = 0.758), and documented thromboembolism occurred in no PBM patients and 2 No-PBM controls (0% vs. 0.33%; *p* = 0.197). Cumulative infection was documented in 26 PBM patients and 25 No-PBM controls (3.40% vs. 4.10%; *p* = 0.566).

Re-exploration for bleeding or cardiac tamponade within 30 days occurred in 9 PBM patients and 5 No-PBM controls (1.18% vs. 0.82%; *p* = 0.596). Cumulative decompensated heart failure through 12 months occurred in 1 PBM patient and 11 No-PBM controls (0.13% vs. 1.80%; *p* = 0.002). Given the sparse event counts, historical comparison, absence of adjustment for multiple testing, and lack of independent event adjudication, these findings were considered descriptive and exploratory.

Among the 355 FCM-treated patients, no myocardial infarction or thromboembolic events were documented through 12 months, while 3 patients experienced stroke. Among the 69 ESA-treated patients, no myocardial infarction, stroke, or thromboembolic events were documented through 12 months. Because FCM and ESA exposures overlapped and treatment allocation was not randomized or adjusted for treatment selection, these descriptive findings do not establish or exclude causal relationships between individual treatments and postoperative events. Detailed interval-specific postoperative safety events and treatment-exposure summaries are presented in Supplementary Table S8.

Hypophosphatemia and adverse effects specifically attributed to FCM or ESA could not be reliably evaluated because serum phosphate measurements and structured treatment-emergent adverse-reaction data were not systematically available.

## 4. Discussion

In this single-center retrospective historical-control cohort of 1374 adults undergoing elective cardiac surgery, a structured multidisciplinary preoperative anemia-management pathway within a Patient Blood Management program was associated with lower RBC transfusion exposure, fewer transfusions of more than two RBC units, shorter postoperative length of stay, and lower estimated direct hospital costs. These associations were observed despite a less favorable measured baseline profile among PBM patients in several respects, including higher prevalences of NYHA class III–IV, ASA class III–IV, moderate renal impairment, and previous cardiac surgery. Conversely, diabetes mellitus was more common in the No-PBM cohort, whereas baseline Hb concentration, detailed procedure distribution, and antithrombotic treatment were similar between groups. The principal transfusion findings remained consistent after conventional multivariable adjustment, propensity-score matching, inverse probability of treatment weighting, and adjacent-period matching. Mortality was low and similar between groups, whereas the association with 12-month readmission was less consistent.

The magnitude of the difference in transfusion exposure was clinically relevant. Any RBC transfusion occurred in 17.3% of PBM patients compared with 39.2% of No-PBM controls, whereas transfusion of more than two RBC units occurred in 2.6% and 15.1%, respectively. After adjustment for age, sex, baseline Hb, procedure type, NYHA functional class, ASA Physical Status, renal function, previous cardiac surgery, diabetes mellitus, anticoagulant use, and antiplatelet therapy, management through the PBM pathway remained associated with lower odds of any RBC transfusion, transfusion of 1–2 units, and transfusion of more than two units, with adjusted ORs of 0.30, 0.49, and 0.14, respectively. EuroSCORE-based models yielded closely concordant estimates.

The association with transfusion of more than two RBC units was also consistent across maximum-likelihood, Firth bias-reduced, propensity-score-matched, and inverse-probability-weighted analyses. The corresponding ORs were 0.14 in the primary model, 0.15 after Firth correction, 0.16 in the EuroSCORE-based model, 0.13 after PSM, and 0.15 after IPTW. Model convergence, low multicollinearity, and stability during leave-one-out refitting reduced concern that this result was driven by separation or influential observations. Nevertheless, this model included only 112 events for 14 predictor parameters, and bootstrap internal validation yielded a calibration slope of approximately 0.72. The precise effect magnitude should therefore be interpreted cautiously, and the discrimination and calibration analyses should be regarded as regression diagnostics rather than as development of a clinical prediction model.

The reduction in transfusion exposure may be clinically relevant beyond blood-product use alone. RBC transfusion after cardiac surgery has been associated with adverse postoperative outcomes in a dose-dependent manner; Lee et al. reported increasing risks of prolonged intubation, infectious complications, renal outcomes, and composite adverse events with greater RBC exposure [8]. However, this observational study cannot establish that reduced transfusion exposure caused the observed differences in postoperative recovery, length of stay, or hospital costs.

The adjacent-period analysis supports the principal findings while reducing, although not eliminating, concern about secular confounding. It yielded 134 adequately balanced pairs of 2018 No-PBM and 2019 PBM patients. Any RBC transfusion occurred in 13.4% of PBM patients and 40.3% of matched No-PBM controls, whereas transfusion of more than two units occurred in 1.5% and 17.2%, respectively. Mean total postoperative LOS was 2.19 days shorter, and mean overall direct hospital cost was EUR 5888 lower, in the 2019 PBM group.

This comparison is particularly informative because institutional guidance during both 2018 and 2019 used the same reference Hb threshold of 9 g/dL for RBC transfusion. The threshold was formally lowered to 8 g/dL in 2020 and therefore applied nominally to the 2022 and 2023 PBM cohorts. Tranexamic acid was also introduced in 2020, and ROTEM use evolved over the study period, becoming consistently integrated into practice only later. Thus, more restrictive transfusion guidance and subsequent perioperative changes could have contributed to the results observed in the later PBM years but cannot by themselves explain the marked association already present in the adjacent 2018–2019 comparison. Nevertheless, transfusion thresholds were not automatic triggers, adoption of practice changes was heterogeneous, and undocumented changes in individual clinical behavior cannot be excluded. The absence of a significant improvement between 2017 and 2018 also provided no evidence of an established downward pre-implementation trend, although two pre-implementation years are insufficient to exclude a longer-term secular trajectory.

Recent matched historical-control data provide additional context for restrictive transfusion practice within cardiac-surgery PBM. Salenger et al. evaluated an ultra-permissive anemia strategy in patients undergoing isolated on-pump CABG, in which RBC transfusion in nonbleeding patients was generally deferred until Hb was <6.0 g/dL in the presence of evidence of inadequate oxygen delivery [22]. Following 1:1 propensity-score matching, the ultra-permissive cohort had substantially lower intraoperative and postoperative RBC use, shorter postoperative length of stay, and no statistically significant increase in operative mortality or stroke. However, that study addressed perioperative tolerance of anemia and transfusion decision-making rather than preoperative anemia correction and, like the present study, used historical controls. Its findings should therefore be considered complementary rather than directly comparable.

The present findings are broadly consistent with previous cardiac-surgery PBM studies. Cahill et al. reported higher day-of-surgery Hb, lower RBC utilization, shorter intensive care and hospital LOS, and lower transfusion-related costs after implementation of a multidisciplinary preoperative anemia-management program [13]. Charbonneau et al. similarly found that systematic correction of anemia and/or iron deficiency using intravenous iron and erythropoietin improved Hb and reduced transfusion requirements [14]. A randomized pilot study by Saour et al. reported lower postoperative RBC exposure with a multimodal strategy including ferric carboxymaltose and erythropoietin [15], whereas the ICARUS study suggested that short-term preoperative iron-deficiency screening and intravenous iron treatment may reduce transfusion and hospital costs [16]. Collectively, these studies support the concept that hematologic optimization may be particularly effective when embedded within a structured multidisciplinary pathway rather than delivered as an isolated pharmacological intervention. This pathway-level interpretation is also consistent with recent calls for closer integration of PBM into enhanced recovery after cardiac surgery pathways, given their shared emphasis on multidisciplinary, preventive, evidence-based, and patient-centered care [23].

A distinctive feature of the present pathway was systematic etiological investigation rather than treatment based solely on Hb concentration. Preoperative anemia was present in 27.7% of PBM patients, and biochemical iron deficiency, irrespective of anemia status, was identified in 26.6%. Iron-deficiency anemia and non-anemic iron deficiency occurred in similar proportions. Folate deficiency was present in 17.1% and vitamin B12 deficiency in 3.3%. These findings demonstrate that potentially correctable abnormalities extended beyond iron deficiency and support comprehensive preoperative hematinic assessment.

The diagnostic strategy incorporated Hb, ferritin, transferrin saturation, reticulocyte hemoglobin content, C-reactive protein, renal function, vitamin B12, and folate, reflecting the heterogeneous causes of anemia in cardiac-surgery patients. This approach is consistent with contemporary recommendations emphasizing early identification and targeted correction of potentially treatable abnormalities [3,6,10,11]. Reticulocyte hemoglobin content may be particularly informative in this setting because it reflects iron immediately available for erythropoiesis and may be less affected than ferritin by inflammatory states [20].

The longitudinal Hb analysis demonstrated a substantial hematological response among treated patients with complete sequential measurements. In 442 patients, mean Hb increased from 12.76 g/dL before treatment to 14.77 g/dL at the first post-treatment reassessment, corresponding to a mean within-patient increase of 2.01 g/dL. At the final preoperative or day-of-surgery assessment, mean Hb was 13.74 g/dL, remaining 0.97 g/dL above the pretreatment concentration but 1.03 g/dL below the first post-treatment value. The median interval from pretreatment assessment to reassessment was 4 weeks and from reassessment to surgery was 6 weeks. The decline before surgery suggests that some of the achieved hematological response may diminish while patients await their procedure and supports coordination between optimization and surgical scheduling. However, these observational data cannot define an optimal or universally applicable waiting interval.

The institution’s use of Hb < 14.5 g/dL as a referral threshold and Hb ≥ 14.5 g/dL as an upper operational optimization goal requires careful interpretation. The value was adopted pragmatically by multidisciplinary consensus to identify patients who might have limited erythrocyte reserve before cardiac procedures involving anticipated blood loss and cardiopulmonary bypass-related hemodilution. The same referral threshold was used in women and men because traditional sex-specific anemia definitions may under-recognize clinically relevant transfusion risk in women undergoing major surgery; women with Hb concentrations of 12.0–12.9 g/dL have been reported to experience greater transfusion exposure than those with Hb ≥ 13 g/dL [12].

However, 14.5 g/dL was not derived from a clinical trial or guideline establishing it as an optimal therapeutic target. Hb < 14.5 g/dL initiated etiological assessment rather than automatic pharmacological treatment, and Hb ≥ 14.5 g/dL represented an upper operational goal when clinically appropriate and feasible rather than a mandatory endpoint. Evidence supporting a sex-neutral Hb threshold of approximately 13 g/dL for defining preoperative anemia should not be conflated with evidence supporting 14.5 g/dL as a universal treatment target. The optimal upper preoperative Hb concentration remains uncertain, and the present study did not compare alternative targets [24].

Treatment was therefore individualized rather than determined solely by the referral threshold. Overall, 442 of 764 PBM patients (57.9%) received pharmacological treatment. FCM was administered to 355 patients (46.5%) and ESA to 69 (9.0%), with 66 patients (8.6%) receiving both; 358 patients (46.9%) received FCM and/or ESA. Folic acid was administered to 131 patients (17.1%) and vitamin B12 to 25 (3.3%), with overlapping exposure categories. The study cannot determine whether pursuit of Hb ≥ 14.5 g/dL provided incremental benefit over a lower individualized target or whether it resulted in additional treatment exposure, cost, or harm. Accordingly, 14.5 g/dL should be interpreted as an institution-specific operational goal rather than as a universally validated therapeutic target.

Importantly, the exposure evaluated in this study was implementation of a bundled PBM pathway rather than administration of any individual treatment. The pathway incorporated screening, standardized etiological assessment, multidisciplinary evaluation, individualized correction of identified abnormalities, repeat Hb reassessment, and coordination with surgical planning. Treatment allocation was not randomized, individual treatment groups overlapped, and treatment selection depended on clinical and laboratory characteristics related to prognosis. Consequently, the observed associations cannot be partitioned into independent effects of FCM, ESA, vitamin supplementation, diagnostic assessment, timing, reassessment, or organizational coordination, and component-specific comparisons would be highly susceptible to confounding by indication.

Management through the PBM pathway was also associated with shorter postoperative hospitalization. Mean total postoperative LOS was 8.05 days in the PBM cohort and 10.07 days in the No-PBM cohort. The association remained after PSM, with a paired mean difference of −2.51 days, and after IPTW, with a weighted mean difference of −1.97 days. These findings are consistent with previous PBM studies reporting reduced intensive care and hospital stay [13,18,22]. Improved erythrocyte reserve and lower transfusion exposure provide plausible explanations, but causal mediation cannot be established from the present data.

The association with 12-month readmission was less robust. Cumulative readmission occurred in 5.4% of PBM patients and 8.0% of No-PBM controls, and lower odds persisted after PSM and IPTW, with ORs of 0.55 and 0.63, respectively. However, the adjacent-period analysis showed no significant difference, with readmission in 4.5% of 2019 PBM patients and 6.0% of matched 2018 controls. Decompensated heart failure was also more frequently documented in the No-PBM cohort and may have contributed to subsequent hospital utilization. Whether this difference was related to postoperative anemia, erythrocyte reserve, underlying ventricular dysfunction, transfusion exposure, or other unmeasured factors cannot be determined. The readmission finding should therefore be considered exploratory.

The economic analysis was intentionally framed as a hospital-perspective cost-consequence analysis rather than as a formal cost-effectiveness evaluation. In the base-case analysis, mean overall direct hospital cost was lower in the PBM cohort, with an unadjusted mean difference of −EUR 4955.78 per patient (bootstrap 95% CI, −EUR 7008.91 to −EUR 3039.86). This association persisted after covariate adjustment: the Gamma model yielded an overall direct-cost ratio of 0.75 (95% CI, 0.68–0.82) and an adjusted marginal mean difference of −EUR 5196 per patient (bootstrap 95% CI, −EUR 6965 to −EUR 3485).

The direction of the economic association was also consistent across sensitivity analyses. The mean difference remained negative after exclusion of readmission costs, propensity-score matching, inverse probability weighting, and adjacent-period matching, and under deterministic ±20% variation in institutional unit costs. The inverse-Gaussian model produced an almost identical overall direct-cost ratio to the Gamma model. These findings indicate that the observed cost association was not dependent on a single modeling assumption, inclusion of readmission expenditure, or the exact institutional unit costs applied in the base case.

Importantly, the economic difference was driven predominantly by hospitalization rather than by lower treatment expenditure. Mean treatment-related cost was similar between cohorts, whereas index-hospitalization expenditure was substantially lower in the PBM group. The findings should therefore be interpreted as institution-specific direct hospital cost consequences rather than as evidence of formal cost-effectiveness, cost utility, return on investment, or societal economic benefit. Patient-level hospital costs of vitamin B12 and folate supplementation were not separately identifiable and may therefore have resulted in modest underestimation of PBM treatment costs.

The findings should also be considered in the context of transfusion stewardship and safety. PBM is intended to reduce avoidable blood exposure rather than eliminate clinically indicated transfusion. Because transfusion appropriateness was not formally adjudicated, the lower rates of RBC transfusion and higher-intensity transfusion should be interpreted as changes in exposure patterns rather than as confirmed improvements in transfusion appropriateness or quality of care. Previous studies have shown that low overall transfusion use does not necessarily imply appropriate transfusion practice and that inappropriate RBC transfusion may carry substantial economic consequences [25,26].

Available postoperative safety events were uncommon. No significant between-cohort differences were observed in myocardial infarction, stroke, thromboembolism, infection, or re-exploration for bleeding or tamponade. Decompensated heart failure was less frequently documented in the PBM cohort, but this exploratory finding was based on few events. Among the 355 FCM-treated patients, three strokes and no myocardial infarction or documented thromboembolic events occurred through 12 months; among the 69 ESA-treated patients, no myocardial infarction, stroke, or documented thromboembolic events were recorded. These treatment-specific observations are descriptive and cannot establish treatment safety because exposures overlapped, treatment allocation was clinically determined, and event numbers were small. Furthermore, serum phosphate and structured treatment-emergent adverse-reaction data were not systematically available, although these represent recognized components of safety assessment for intravenous iron and ESA therapy [27,28]. The present study therefore cannot establish the treatment-specific safety of FCM or ESA.

This study has several strengths. It includes a relatively large real-world cardiac-surgery cohort with individual patient-level clinical and economic data, examines both transfusion exposure and transfusion intensity, and evaluates postoperative LOS, readmission, mortality, safety events, and direct hospital costs. Longitudinal Hb measurements were available at three clinically relevant time points in 442 treated patients. Analytical robustness was explored using conventional and EuroSCORE-based regression, Firth correction, leave-one-out analysis, propensity-score matching, IPTW, an adjacent-period matched comparison, bootstrap methods, and alternative economic model specifications.

Several limitations remain important. First, the historical-control design is the principal limitation and prevents causal attribution. The groups occupied non-overlapping calendar periods, and transfusion guidance and perioperative practice evolved over time. Although the adjacent-period 2018–2019 analysis reproduced the principal transfusion, LOS, and cost associations under the same 9 g/dL transfusion guidance and before the introduction of tranexamic acid, it cannot exclude undocumented practice changes. PSM and IPTW address measured differences but cannot account for unmeasured secular changes, and exclusion of the pandemic-affected years 2020 and 2021 further limited assessment of temporal evolution.

Selection bias is also possible because incomplete principal-outcome data led to exclusion of 44 of 654 historical patients (6.7%) compared with 6 of 770 PBM patients (0.8%). Historical documentation was less standardized, and the direction of any resulting bias is uncertain. Propensity methods cannot correct selection occurring before formation of the analytical cohort.

Residual confounding from incompletely measured comorbidity and operative complexity also remains possible. Left ventricular ejection fraction was not systematically available, and NYHA class cannot fully characterize ventricular dysfunction, pulmonary hypertension, valvular severity, or heart-failure phenotype. Other potentially relevant factors, including frailty, chronic pulmonary and vascular disease, body mass index, coagulation variables, bypass and cross-clamp duration, quantified blood loss, number of grafts or valves treated, nadir hematocrit, and patient-level use of tranexamic acid, cell salvage, or ROTEM, were not consistently harmonized across periods. Similarly, hematinic abnormalities could not be used as common adjustment variables because they were not systematically investigated in historical controls.

The direction of residual confounding could plausibly operate in either direction. Greater unmeasured cardiac dysfunction, frailty, pulmonary hypertension, or operative complexity in the PBM cohort—consistent with its higher observed NYHA and ASA classifications and greater frequency of previous cardiac surgery—could attenuate an association favoring PBM. Conversely, greater unrecognized hematinic abnormalities in historical controls, more restrictive later transfusion behavior, or greater use of blood-conservation measures during PBM years could exaggerate the association. The large and concordant transfusion findings across analytical approaches reduce, but do not eliminate, this concern. LOS and cost are likely more susceptible to unmeasured operative and organizational factors, whereas the 12-month readmission association is particularly uncertain because it was not reproduced in the adjacent-period analysis.

The longitudinal Hb analysis was restricted to treated PBM patients with complete sequential measurements and therefore remains susceptible to treatment-selection and measurement-availability bias. Historical controls lacked corresponding serial measurements. The study also evaluated a bundled pathway rather than individual components, preventing estimation of the independent efficacy of FCM, ESA, vitamin supplementation, diagnostic assessment, or organizational features. For the >2-unit transfusion model, the relatively low event-to-parameter ratio and evidence of model optimism warrant caution regarding the precise OR despite concordant sensitivity analyses.

Safety assessment was retrospective, event counts were small, and events were not independently adjudicated. Absence of significant differences should not be interpreted as proof of equivalence or treatment safety. Serum phosphate and structured FCM- and ESA-specific adverse-event information were incomplete. In addition, transfusion appropriateness was not adjudicated, and non-RBC blood products were not principal study outcomes.

Finally, the economic estimates reflect one Portuguese hospital and a common 2024 institutional unit-cost schedule. Hospitalization was valued using setting-specific daily costs rather than comprehensive patient-level microcosting, and several cost domains were not captured, including detailed diagnostic, procedural, outpatient, rehabilitation, societal, and longer-term costs. Vitamin B12 and folate costs were also not separately identifiable. These findings therefore describe direct hospital cost consequences within the observed 12-month horizon and should not be generalized to long-term payer or societal cost-effectiveness.

Overall, the consistency of the transfusion, LOS, and cost associations across conventional, propensity-based, and adjacent-period analyses supports the robustness of the pathway-level findings to measured baseline differences. However, the historical-control design and potential residual clinical and secular confounding preclude causal attribution. The less consistent readmission finding and limited treatment-specific safety data warrant additional caution. Prospective multicenter studies with contemporaneous controls should evaluate alternative preoperative Hb targets, phenotype-specific treatment responses, durability of Hb optimization, treatment-related safety, postoperative anemia recovery, patient-centered outcomes, and formal cost-effectiveness.

## 5. Conclusions

In this single-center retrospective historical-control cohort study, implementation of a structured multidisciplinary preoperative anemia-management pathway within a Patient Blood Management program was associated with lower RBC transfusion exposure, fewer transfusions of more than two RBC units, shorter postoperative length of stay, and lower estimated direct hospital costs among adults undergoing elective cardiac surgery. The principal transfusion associations were consistent across conventional multivariable, penalized, propensity-score-matched, inverse-probability-weighted, and adjacent-period analyses. Length-of-stay and cost findings were likewise concordant across propensity-based and adjacent-period analyses.

Mortality was low and similar between cohorts, whereas the association with lower 12-month readmission was less consistent and should be interpreted cautiously. Recorded major postoperative safety events were uncommon; however, the retrospective design and incomplete treatment-specific adverse-event ascertainment preclude definitive conclusions regarding the safety of FCM or ESA.

The economic findings represent institution-specific direct hospital cost consequences and should not be interpreted as evidence of formal cost-effectiveness, cost-utility, return on investment, or societal economic benefit. More broadly, the historical-control design, non-overlapping calendar periods, and potential residual clinical and secular confounding prevent causal attribution. These findings should therefore be interpreted as pathway-level associations rather than as evidence of a causal effect of the PBM pathway or any individual treatment component.

Prospective multicenter studies using contemporaneous control groups are needed to confirm these findings and to define optimal preoperative Hb targets, phenotype-specific treatment strategies, durability of Hb optimization, treatment-related safety, patient-centered outcomes, and formal cost-effectiveness.

## Figures and Tables

**Table 1:** Baseline characteristics of the complete study cohorts before adjustment.

Variable	PBM Cohort (*n* = 764)	No-PBM Cohort (*n* = 610)	*p*-Value	Absolute SMD
Age, years	67.96 ± 9.52	67.87 ± 11.13	0.881	0.008
Male sex, *n* (%)	478 (62.6%)	390 (63.9%)	0.601	0.028
Baseline Hb, g/dL	13.43 ± 1.35	13.50 ± 1.70	0.444	0.042
Detailed procedure type, *n* (%)			0.415	
CABG-containing procedures	223 (29.2%)	196 (32.1%)	—	0.064
Aortic valve surgery	264 (34.6%)	204 (33.4%)	—	0.023
Mitral or tricuspid valve surgery	31 (4.1%)	21 (3.4%)	—	0.032
Ascending aortic surgery	19 (2.5%)	12 (2.0%)	—	0.035
Combined non-CABG procedures	203 (26.6%)	167 (27.4%)	—	0.018
Atrial septal defect repair	24 (3.1%)	10 (1.6%)	—	0.098
NYHA class III–IV, *n* (%)	242 (31.7%)	93 (15.2%)	<0.001	0.395
ASA class III–IV, *n* (%)	749 (98.0%)	565 (92.6%)	<0.001	0.259
eGFR category, mL/min/1.73 m^2^, *n* (%)			<0.001	
<30	17 (2.2%)	16 (2.6%)	—	0.026
30–60	155 (20.3%)	55 (9.0%)	—	0.323
>60	592 (77.5%)	539 (88.4%)	—	0.292
Previous cardiac surgery, *n* (%)	30 (3.9%)	6 (1.0%)	<0.001	0.191
Diabetes mellitus, *n* (%)	195 (25.5%)	198 (32.5%)	0.005	0.153
Preoperative anticoagulant use, *n* (%)	112 (14.7%)	108 (17.7%)	0.126	0.083
Preoperative antiplatelet therapy, *n* (%)	233 (30.5%)	179 (29.3%)	0.643	0.025
EuroSCORE II, %	2.16 ± 2.80	1.93 ± 1.48	0.055	0.101
EuroSCORE II > 2%, *n* (%)	233 (30.5%)	171 (28.0%)	0.319	0.054

Note: Data are presented as mean ± standard deviation or *n* (%). Percentages were calculated using the total number of patients in each cohort. Continuous variables were compared using independent-samples Student’s *t*-tests or Welch’s *t*-tests, and categorical variables using the chi-square test or Fisher’s exact test, as appropriate. For detailed procedure type and eGFR category, *p*-values represent omnibus comparisons across all categories; individual category rows were not tested separately. Standardized mean differences are presented as absolute values, with values ≥ 0.10 indicating potentially meaningful baseline imbalance. The six procedure categories were used in the propensity-score analyses, whereas procedure type was consolidated into three categories for the conventional multivariable regression models, as described in Section 2.2. ASA, American Society of Anesthesiologists Physical Status; CABG, coronary artery bypass grafting; eGFR, estimated glomerular filtration rate; EuroSCORE II, European System for Cardiac Operative Risk Evaluation II; Hb, hemoglobin; NYHA, New York Heart Association; No-PBM, historical control cohort not managed through the structured Patient Blood Management pathway; PBM, Patient Blood Management; SMD, standardized mean difference.

**Table 2:** Preoperative anemia phenotypes and hematinic abnormalities in the PBM cohort.

Condition	PBM Cohort (*n* = 764), *n* (%)
Preoperative anemia	212 (27.7%)
Biochemical iron deficiency, irrespective of anemia status	203 (26.6%)
Iron-deficiency anemia	101 (13.2%)
Non-anemic iron deficiency	102 (13.4%)
Anemia with inflammation	28 (3.7%)
Probable anemia of chronic disease	8 (1.0%)
Mixed iron-deficiency and inflammatory anemia	20 (2.6%)
Anemia with severe renal impairment	5 (0.7%)
Folate deficiency	131 (17.1%)
Vitamin B12 deficiency	25 (3.3%)

Note: Data are presented as *n* (%), using the full PBM cohort (*n* = 764) as the denominator. Overall categories may overlap; indented subcategories are mutually exclusive within their respective parent categories. Preoperative anemia was defined as Hb < 13 g/dL. Biochemical iron deficiency was defined as ferritin < 30 ng/mL, TSAT < 20%, or CHr < 29.1 pg. Anemia with inflammation was defined as Hb < 13 g/dL and CRP > 10 mg/L; patients with concomitant biochemical iron deficiency were classified as having mixed iron-deficiency and inflammatory anemia, whereas those without biochemical iron deficiency were classified as having probable ACD. Folate deficiency was defined as serum folate < 2 ng/mL, vitamin B12 deficiency as serum vitamin B12 < 200 pg/mL, and preoperative anemia with severe renal impairment as Hb < 13 g/dL with eGFR < 30 mL/min/1.73 m^2^. The renal category was descriptive and did not establish severe renal impairment as the sole or confirmed cause of anemia. ACD, anemia of chronic disease; CHr, reticulocyte hemoglobin content; CRP, C-reactive protein; eGFR, estimated glomerular filtration rate; Hb, hemoglobin; PBM, Patient Blood Management; TSAT, transferrin saturation.

**Table 3:** Length of stay during the index hospitalization and subsequent readmissions, by study cohort.

Outcome	PBM Cohort	No-PBM Cohort	*p*-Value
T-SICU LOS, days	2.88 (2.70–3.08)	3.90 (3.55–4.57)	<0.001
TSU LOS, days	5.17 (4.87–5.49)	6.17 (5.71–6.80)	0.002
Total postoperative LOS, days	8.05 (7.64–8.50)	10.07 (9.42–10.97)	<0.001
Readmission T-SICU LOS, days	0.68 (0.38–1.23)	2.56 (1.60–4.18)	0.006
Readmission TSU LOS, days	14.60 (11.08–19.32)	11.09 (8.44–14.38)	0.177
Total readmission LOS, days	15.28 (11.77–20.02)	13.64 (10.89–17.09)	0.533

Note: Data are presented as mean (95% confidence interval). Index-hospitalization analyses included all patients in each cohort. Readmission LOS analyses included only patients with at least one readmission within 12 months after discharge from the index hospitalization (PBM, *n* = 41; No-PBM, *n* = 49). Between-cohort comparisons were performed using independent-samples Student’s *t*-tests or Welch’s *t*-tests, as appropriate. LOS, length of stay; No-PBM, historical control cohort not managed through the structured Patient Blood Management pathway; PBM, Patient Blood Management; T-SICU, thoracic surgery intensive care unit; TSU, thoracic surgery unit.

**Table 4:** Direct hospital costs per patient in the PBM and No-PBM groups.

Cost Category	PBM Cohort, Mean ± SD; Median (IQR)	No-PBM Cohort, Mean ± SD; Median (IQR)	Mean Difference, PBM Minus No-PBM (Bootstrap 95% CI)
Ferric carboxymaltose	81.72 ± 89.68; 0 (0–174.40)	0; 0 (0–0)	81.72 (75.10 to 88.34)
Erythropoiesis-stimulating agent	11.05 ± 40.39; 0 (0–0)	0; 0 (0–0)	11.05 (8.47 to 14.00)
RBC transfusion	30.82 ± 83.26; 0 (0–0)	114.79 ± 240.78; 0 (0–104.20)	−83.97 (−105.25 to −64.98)
Treatment-related cost	123.60 ± 139.89; 104.20 (0–174.40)	114.79 ± 240.78; 0 (0–104.20)	8.81 (−11.60 to 28.98)
Index hospitalization	14,419.06 ± 11,366.94; 12,003 (9469–14,537)	18,863.40 ± 19,734.34; 13,270 (10,736–19,779.75)	−4444.34 (−6339.47 to −2670.26)
Readmissions within 12 months	1089.94 ± 5866.30; 0 (0–0)	1610.19 ± 7161.90; 0 (0–0)	−520.25 (−1258.54 to 186.66)
Total hospitalization cost	15,509.00 ± 13,499.03; 12,003 (9469–15,137)	20,473.59 ± 21,759.68; 13,720 (10,736–21,397)	−4964.59 (−6988.04 to −3120.12)
Overall direct hospital cost	15,632.60 ± 13,507.18; 12,003 (9643.40–15,311.40)	20,588.38 ± 21,904.36; 13,870 (10,736–21,628.30)	−4955.78 (−7008.91 to −3039.86)

Note: Costs are presented in 2024 euros as mean ± standard deviation and median (interquartile range). Mean differences were calculated as PBM minus No-PBM, with negative values indicating lower mean costs in the PBM cohort. Confidence intervals are percentile intervals obtained from 1000 stratified nonparametric bootstrap resamples. All resource quantities were valued using the same 2024 institutional unit-cost schedule. Index-hospitalization cost comprised T-SICU and TSU costs during the index admission; total hospitalization cost comprised index-hospitalization and readmission costs; and overall direct hospital cost comprised treatment-related and total hospitalization costs. IQR, interquartile range; No-PBM, historical control cohort not managed through the structured Patient Blood Management pathway; PBM, Patient Blood Management; RBC, red blood cell; SD, standard deviation.

**Table 5:** Association between management through the PBM pathway and RBC transfusion outcomes in crude and expanded multivariable models.

Outcome	Crude OR (95% CI)	*p*	Individual-Covariate aOR (95% CI)	*p*	EuroSCORE-Based aOR (95% CI)	*p*
Any RBC transfusion	0.32 (0.25–0.42)	<0.001	0.30 (0.23–0.39)	<0.001	0.32 (0.25–0.42)	<0.001
1–2 RBC units	0.54 (0.41–0.71)	<0.001	0.49 (0.37–0.66)	<0.001	0.53 (0.40–0.70)	<0.001
>2 RBC units	0.15 (0.09–0.25)	<0.001	0.14 (0.08–0.24)	<0.001	0.16 (0.09–0.26)	<0.001

Note: The No-PBM cohort was the reference group. The primary individual-covariate model included age, sex, baseline Hb concentration, procedure type, NYHA functional class, ASA Physical Status category, eGFR category, previous cardiac surgery, diabetes mellitus, preoperative anticoagulant use, and antiplatelet therapy. Procedure type was grouped as CABG-containing procedures, isolated valve surgery, or other non-CABG procedures. The EuroSCORE-based sensitivity model included continuous EuroSCORE II, baseline Hb concentration, procedure type, ASA Physical Status category, preoperative anticoagulant use, and antiplatelet therapy. All analyses included 1374 patients. aOR, adjusted odds ratio; ASA, American Society of Anesthesiologists Physical Status; CABG, coronary artery bypass grafting; CI, confidence interval; eGFR, estimated glomerular filtration rate; Hb, hemoglobin; No-PBM, historical control cohort not managed through the structured Patient Blood Management pathway; NYHA, New York Heart Association; OR, odds ratio; PBM, Patient Blood Management; RBC, red blood cell.

**Table 6:** Principal clinical and economic outcomes after propensity-score matching and inverse probability of treatment weighting. (**A**) Panel A. Propensity-score-matched analysis. (**B**) Inverse-probability-of-treatment-weighted analysis.

(**A**)				
**Outcome**	**PBM Cohort** **(*n* = 519)**	**No-PBM Cohort** **(*n* = 519)**	**Matched Estimate** **(95% CI)**	***p-*** **Value**
Any RBC transfusion, *n* (%)	86 (16.6%)	208 (40.1%)	OR 0.29 (0.21–0.39)	<0.001
Transfusion of 1–2 RBC units, *n* (%)	74 (14.3%)	127 (24.5%)	OR 0.50 (0.36–0.69)	<0.001
Transfusion of >2 RBC units, *n* (%)	12 (2.3%)	81 (15.6%)	OR 0.13 (0.07–0.24)	<0.001
RBC units transfused, mean	0.28	1.13	MD −0.85 (−1.07 to −0.63)	<0.001
T-SICU LOS, days, mean	2.66	4.01	MD −1.34 (−1.91 to −0.77)	<0.001
TSU LOS, days, mean	5.07	6.24	MD −1.17 (−1.86 to −0.49)	<0.001
Total postoperative LOS, days, mean	7.74	10.25	MD −2.51 (−3.47 to −1.56)	<0.001
Readmission within 12 months, *n* (%)	24 (4.6%)	43 (8.3%)	OR 0.55 (0.33–0.91)	0.025
Overall direct hospital cost, EUR, mean	14,850	21,051	MD −6201 (−8481 to −4195)	—
(**B**)				
**Outcome**	**PBM Weighted Estimate**	**No-PBM Weighted Estimate**	**Weighted Estimate (95% CI)**	
Any RBC transfusion	16.7%	39.6%	OR 0.31 (0.24–0.39)	
1–2 RBC units	14.1%	24.5%	OR 0.50 (0.38–0.66)	
>2 RBC units	2.6%	15.1%	OR 0.15 (0.09–0.25)	
RBC units transfused	0.29	1.14	MD −0.85 (−1.09 to −0.60)	
T-SICU LOS	2.77	3.87	MD −1.11 (−1.56 to −0.65)	
TSU LOS	5.24	6.10	MD −0.86 (−1.48 to −0.24)	
Total postoperative LOS	8.01	9.97	MD −1.97 (−2.81 to −1.12)	
Readmission within 12 months	4.9%	7.6%	OR 0.63 (0.41–0.99)	
Overall direct hospital cost, EUR	15,457	20,351	MD −4894 (−6735 to −3206)	

Note: Panel A presents results from the 1:1 propensity-score-matched population; binary outcomes were analyzed using conditional logistic regression and continuous outcomes using paired comparisons. Panel B presents stabilized inverse-probability-of-treatment-weighted estimates; binary outcomes were analyzed using weighted logistic regression and continuous outcomes using weighted linear regression, both with robust sandwich standard errors. Effect estimates are expressed as odds ratios for binary outcomes and mean differences for continuous outcomes. Mean differences were calculated as PBM minus No-PBM; negative values therefore indicate lower values in the PBM cohort. The 95% CI for the PSM cost difference was obtained by nonparametric bootstrap resampling of matched pairs, whereas the IPTW cost CI was obtained from 1000 stratified bootstrap resamples with re-estimation of the propensity-score model and stabilized weights. No *p* values are reported for economic comparisons because uncertainty was quantified using bootstrap confidence intervals. CI, confidence interval; IPTW, inverse probability of treatment weighting; LOS, length of stay; MD, mean difference; No-PBM, historical control cohort not managed through the structured Patient Blood Management pathway; OR, odds ratio; PBM, Patient Blood Management; PSM, propensity-score matching; RBC, red blood cell; T-SICU, thoracic surgery intensive care unit; TSU, thoracic surgery unit.

**Table 7:** Available postoperative safety events in the PBM and No-PBM cohorts.

Safety Outcome	PBM ≤ 30 Days, *n* (%)	No-PBM ≤ 30 Days, *n* (%)	*p*-Value	PBM Cumulative 12 Months, *n* (%)	No-PBM Cumulative 12 Months, *n* (%)	*p*-Value
Myocardial infarction	0 (0.00%)	2 (0.33%)	0.197	1 (0.13%)	2 (0.33%)	0.588
Stroke	4 (0.52%)	2 (0.33%)	0.699	5 (0.65%)	5 (0.82%)	0.758
Thromboembolism	0 (0.00%)	1 (0.16%)	0.444	0 (0.00%)	2 (0.33%)	0.197
Infection	25 (3.27%)	12 (1.97%)	0.179	26 (3.40%)	25 (4.10%)	0.566
Re-exploration for bleeding or tamponade	9 (1.18%)	5 (0.82%)	0.596	9 (1.18%)	5 (0.82%)	0.596
Decompensated heart failure	0 (0.00%)	7 (1.15%)	0.003	1 (0.13%)	11 (1.80%)	0.002

Note: Data are presented as *n* (%). Cumulative 12-month incidence represents occurrence of the event within 30 days or from >30 days to 12 months after surgery. Between-cohort comparisons were performed using two-sided Fisher’s exact tests because of the small event counts. Analyses were exploratory, with no adjustment for multiple comparisons. Events were identified retrospectively from available clinical records and were not independently adjudicated. These postoperative events should not be interpreted as adverse reactions causally attributable to FCM, ESA, or any individual component of the PBM pathway. ESA, erythropoiesis-stimulating agent; FCM, ferric carboxymaltose; No-PBM, historical control cohort not managed through the structured Patient Blood Management pathway; PBM, Patient Blood Management.

## Data Availability

The patient-level data analyzed in this study are available from the corresponding author upon reasonable request, subject to institutional ethics approval and applicable privacy and data-protection requirements. The patient-level data are not publicly available because of privacy and ethical restrictions.

## Supplementary Materials

The following supporting information can be downloaded at https://www.mdpi.com/article/10.3390/jcm15187150/s1. Figure S1: Covariate balance before and after propensity-score matching and inverse probability of treatment weighting in the complete study cohort; Figure S2: Covariate balance before and after adjacent-period matching of 2018 No-PBM controls and 2019 PBM patients; Figure S3: Internal calibration of the expanded individual-covariate logistic-regression model for transfusion of >2 RBC units; Table S1: Baseline covariate balance before and after propensity-score matching and inverse probability of treatment weighting in the complete study cohort; Table S2: Pre-implementation temporal comparison of clinical and economic outcomes in 2017 and 2018; Table S3: Baseline covariate balance before and after adjacent-period matching of 2018 No-PBM controls and 2019 PBM patients; Table S4: Clinical and economic outcomes in the adjacent-period matched analysis of 2018 No-PBM controls and 2019 PBM patients; Table S5: Full coefficient estimates from the expanded individual-covariate and EuroSCORE-based logistic-regression models for RBC transfusion outcomes; Table S6: Event-to-variable ratios, convergence, influence, multicollinearity, and internal-validation diagnostics for the expanded logistic-regression models; Table S7: Sensitivity analyses of overall direct hospital cost; Table S8: Interval-specific postoperative safety events and descriptive event counts among FCM-treated and ESA-treated patients.

## Abbreviations

The following abbreviations are used in this manuscript:

ACD	Anemia of chronic disease
ASA	American Society of Anesthesiologists
AUC	Area under the receiver operating characteristic curve
CABG	Coronary artery bypass grafting
CHr	Reticulocyte hemoglobin content
CI	Confidence interval
COVID-19	Coronavirus disease 2019
CRP	C-reactive protein
eGFR	Estimated glomerular filtration rate
ESA	Erythropoiesis-stimulating agent
EuroSCORE II	European System for Cardiac Operative Risk Evaluation II
FCM	Ferric carboxymaltose
Hb	Hemoglobin
ICU	Intensive care unit
IDA	Iron-deficiency anemia
IPTW	Inverse probability of treatment weighting
IU	International units
LOS	Length of stay
MIDCAB	Minimally invasive direct coronary artery bypass
NYHA	New York Heart Association
No-PBM	Non-Patient Blood Management group (historical control cohort not managed through the structured Patient Blood Management pathway)
OR	Odds ratio
PBM	Patient Blood Management
PSM	Propensity-score matching
RBC	Red blood cell
ROTEM	Rotational thromboelastometry
SMD	Standardized mean differences
T-SICU	Thoracic surgery intensive care unit
TSAT	Transferrin saturation
TSU	Thoracic surgery unit
WHO	World Health Organization
