# Supplementary material for: Clinical and Economic Outcomes Associated with Structured Preoperative Anemia Management in Elective Cardiac Surgery: A Patient Blood Management Cohort Study
Source: J Clin Med. 2026 Sep 15;15(18):7150. doi: 10.3390/jcm15187150 (PMC13607426; doi:10.3390/jcm15187150)
Supplement: Supplementary file 1 [file jcm-15-07150-s001.zip › jcm-4479004-supplementary.pdf]

Journal of
Clinical Medicine

MDPI

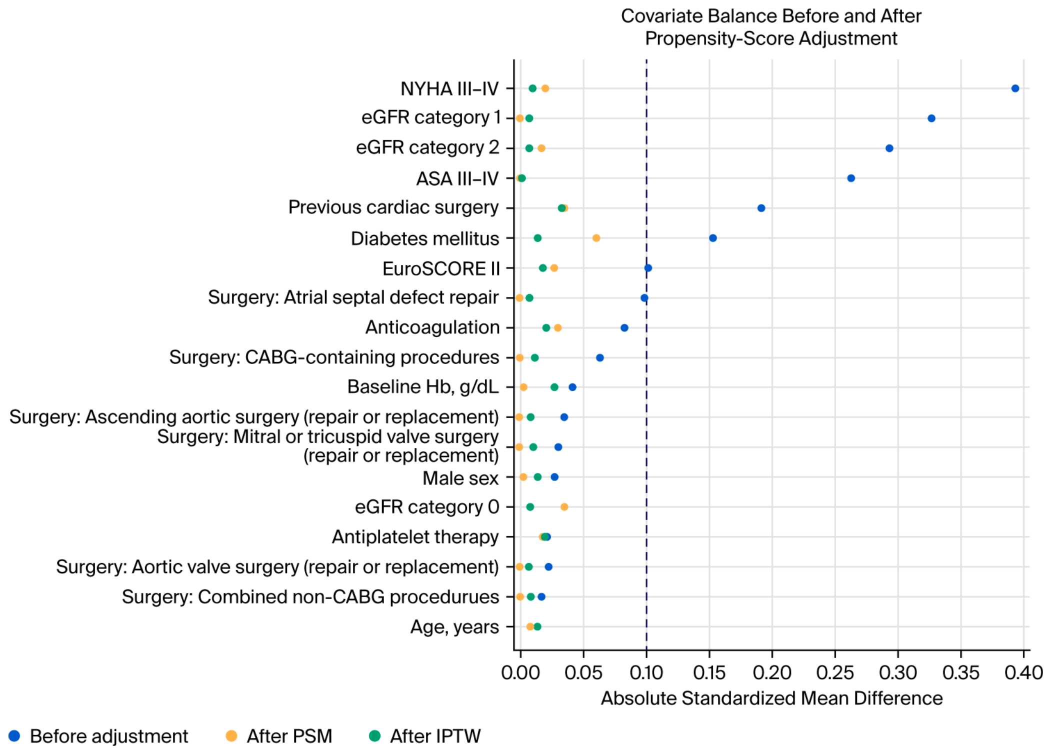

Figure S1. Covariate balance before and after propensity-score matching and inverse probability of treatment weighting in the complete study cohort. Absolute standardized mean differences are shown before adjustment, after 1:1 propensity-score matching, and after stabilized inverse probability of treatment weighting. The dashed vertical line at 0.10 indicates the prespecified threshold for acceptable covariate balance. EuroSCORE II was not included in the propensity-score model and is shown as an additional balance diagnostic. IPTW, inverse probability of treatment weighting; PSM, propensity-score matching; SMD, standardized mean difference. ASA, American Society of Anesthesiologists physical status; CABG, coronary artery bypass grafting; eGFR, estimated glomerular filtration rate; Hb, hemoglobin; IPTW, inverse probability of treatment weighting; NYHA, New York Heart Association; PSM, propensity-score matching; SMD, standardized mean difference.

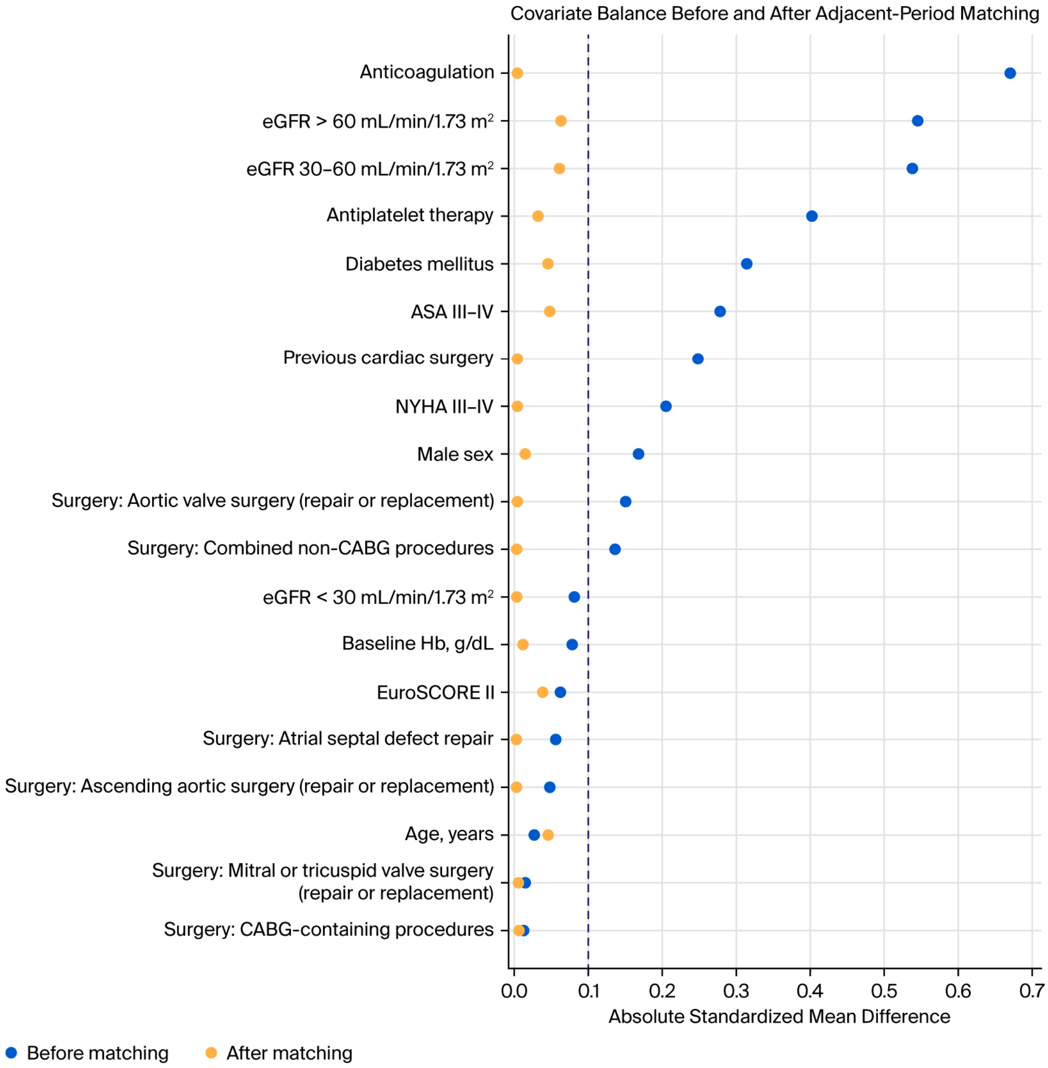

Figure S2. Covariate balance before and after adjacent-period matching of 2018 No-PBM controls and 2019 PBM patients. Absolute standardized mean differences are presented before and after 1:1 matching. The adjacent-period analysis yielded 134 matched pairs. Matching was performed without replacement, with exact matching on detailed surgical category, NYHA functional class, and previous cardiac surgery; Mahalanobis distance within the propensity-score caliper was used to optimize balance across the remaining baseline covariates. The dashed vertical line indicates the prespecified threshold of 0.10 for acceptable covariate balance. ASA, American Society of Anesthesiologists Physical Status; CABG, coronary artery bypass grafting; eGFR, estimated glomerular filtration rate; Hb, hemoglobin; No-PBM, historical control cohort not managed through the structured Patient Blood Management pathway; NYHA, New York Heart Association; PBM, Patient Blood Management; SMD, standardized mean difference.

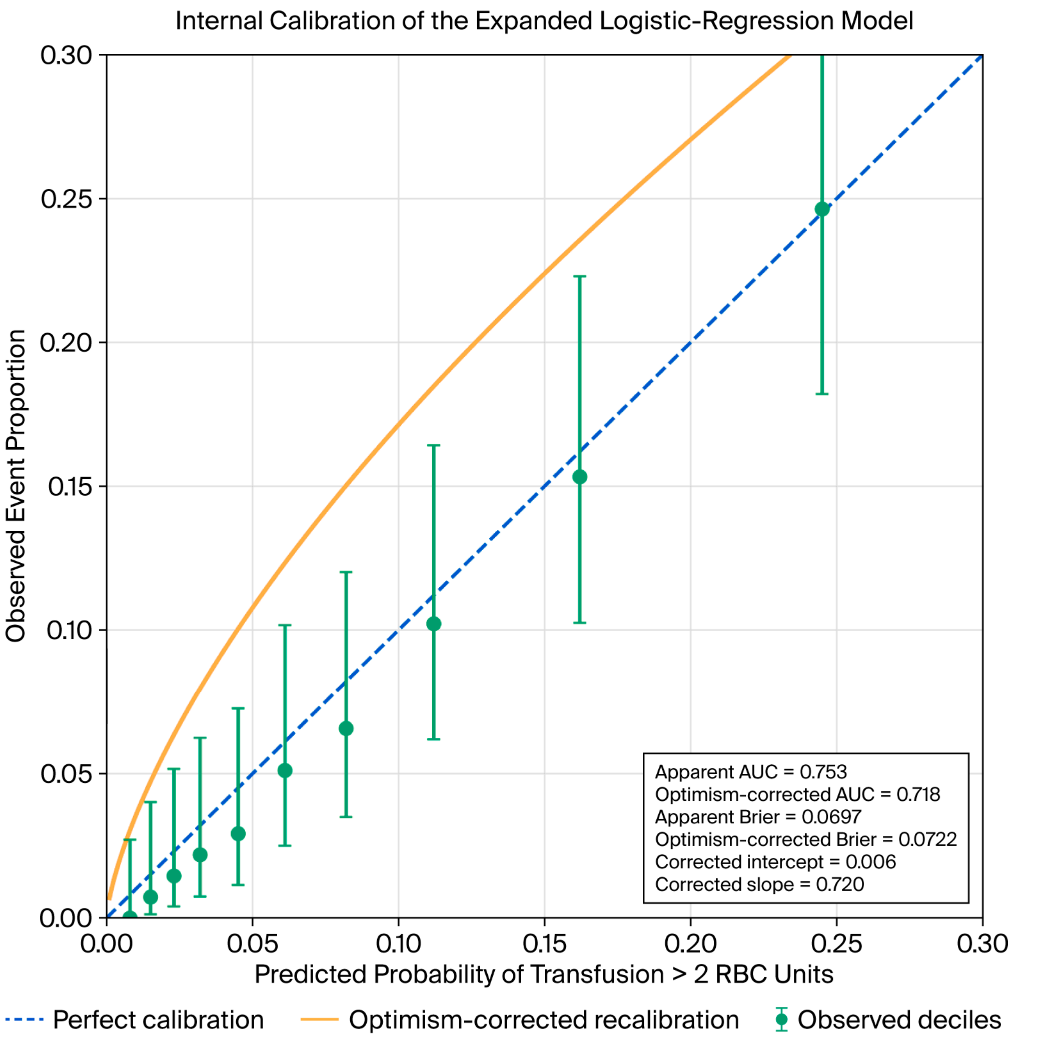

Figure S3. Internal calibration of the expanded individual-covariate logistic regression model for transfusion of >2 RBC units. Circles represent observed event proportions within deciles of predicted risk, with Wilson 95% confidence intervals. The dashed diagonal line indicates perfect calibration. The solid curve represents the bootstrap optimism-corrected logistic recalibration curve, derived from the corrected joint calibration intercept and slope after 1000 bootstrap resamples. The apparent and optimism-corrected areas under the receiver operating characteristic curve were 0.753 and 0.718, respectively, and the corresponding Brier scores were 0.0697 and 0.0722. The optimism-corrected calibration-in-the-large was 0.006, and the optimism-corrected calibration slope was 0.720. These measures were used as internal regression-model diagnostics and do not constitute external validation of a clinical prediction model. CI, confidence interval; RBC, red blood cell.

Table S1. Baseline covariate balance before and after propensity-score matching and inverse probability of treatment weighting in the complete study cohort.

Covariate	Before Adjustment ∣SMD∣	After PSM ∣SMD∣	After IPTW ∣SMD∣
Age, years	0.008	0.009	0.014
Male sex	0.028	0.004	0.014
Baseline Hb, g/dL	0.042	0.004	0.028
Aortic valve surgery	0.023	0.000	0.008
Ascending aortic surgery	0.035	0.000	0.009
Atrial septal defect repair	0.098	0.000	0.008
CABG-containing procedures	0.064	0.000	0.012
Combined non-CABG procedures	0.018	0.000	0.009

https://doi.org/10.3390/jcm15187150

Mitral or tricuspid valve surgery	0.032	0.000	0.011
NYHA class III–IV	0.395	0.020	0.011
ASA class III–IV	0.259	0.000	0.002
eGFR < 30 mL/min/1.73 m^2	0.026	0.035	0.008
eGFR 30–60 mL/min/1.73 m^2	0.323	0.000	0.008
eGFR > 60 mL/min/1.73 m^2	0.292	0.017	0.008
Previous cardiac surgery	0.191	0.036	0.034
Diabetes mellitus	0.153	0.059	0.015
Preoperative anticoagulant use	0.083	0.030	0.021
Preoperative antiplatelet therapy	0.025	0.017	0.020
EuroSCORE II, % *	0.101	0.027	0.018

Note: Absolute standardized mean differences (|SMD|) are reported; values < 0.10 were considered indicative of acceptable covariate balance. PSM used 1:1 optimal matching without replacement, exact matching on the six-category surgical classification, and a caliper of 0.2 standard deviations of the logit of the propensity score. IPTW used stabilized weights targeting the average treatment effect. * Continuous EuroSCORE II was not included in the propensity-score model because of overlap with several component clinical risk variables; it was evaluated as an additional balance diagnostic.

Table S2. Pre-implementation temporal comparison of clinical and economic outcomes in 2017 and 2018.

Outcome	2017 No-PBM (n = 312)	2018 No-PBM (n = 298)	p-Value or Mean Difference (95% CI)
Any RBC transfusion	114 (36.5%)	125 (41.9%)	0.171
Transfusion of 1–2 RBC units	71 (22.8%)	76 (25.5%)	0.428
Transfusion of > 2 RBC units	43 (13.8%)	49 (16.4%)	0.359
RBC units transfused, mean	0.96	1.25	0.128
T-SICU LOS, days, mean	3.87	3.92	0.915
TSU LOS, days, mean	6.39	5.94	0.412
Total postoperative LOS, days, mean	10.27	9.86	0.603
Readmission within 12 months	23 (7.4%)	26 (8.7%)	0.539
All-cause mortality within 12 months	2 (0.6%)	2 (0.7%)	1.000
Index-hospitalization cost, EUR, mean	19,074	18,643	MD −431 (−3427 to 3023)
Total hospitalization cost, EUR, mean	20,344	20,609	MD 264 (−3059 to 3958)
Overall direct hospital cost, EUR, mean	20,445	20,739	MD 294 (−3057 to 4035)

Note: Categorical outcomes are presented as n (%), and continuous clinical outcomes as means. Categorical comparisons used the chi-square test or Fisher exact test, as appropriate; continuous non-cost outcomes used independent-samples t tests (Welch correction when required). Cost differences are calculated as 2018 minus 2017 and are accompanied by percentile 95% confidence intervals from 1000 stratified nonparametric bootstrap resamples. No statistically significant pre-implementation improvement was identified in the principal transfusion, length-of-stay, readmission, mortality, or direct-cost outcomes. CI, confidence interval; LOS, length of stay; MD, mean difference; RBC, red blood cell; T-SICU, thoracic surgery intensive care unit; TSU, thoracic surgery unit.

Table S3. Baseline covariate balance before and after adjacent-period matching of 2018 No-PBM controls and 2019 PBM patients.

Covariate	Before Matching |SMD|	After Matching |SMD|
Age, years	0.029	0.045
Male sex	0.168	0.014
Baseline Hb, g/dL	0.079	0.011
Aortic valve surgery	0.149	0.000
Ascending aortic surgery	0.050	0.000

Atrial septal defect repair	0.058	0.000
CABG-containing procedures	0.011	0.000
Combined non-CABG procedures	0.137	0.000
Mitral or tricuspid valve surgery	0.013	0.000
NYHA class III–IV	0.206	0.000
ASA class III–IV	0.279	0.047
eGFR < 30 mL/min/1.73 m^2	0.080	0.000
eGFR 30–60 mL/min/1.73 m^2	0.540	0.060
eGFR > 60 mL/min/1.73 m^2	0.548	0.063
Previous cardiac surgery	0.250	0.000
Diabetes mellitus	0.314	0.045
Preoperative anticoagulant use	0.671	0.000
Preoperative antiplatelet therapy	0.402	0.031
EuroSCORE II, % *	0.062	0.037

Note: Absolute standardized mean differences (|SMD|) are reported; values < 0.10 were considered indicative of acceptable balance. The adjacent-period propensity score was re-estimated in the 2018–2019 population using the same baseline covariates as the primary propensity-score model. Matching was 1:1 without replacement, with exact matching on detailed surgical category, NYHA category, and previous cardiac surgery, and Mahalanobis distance within the propensity-score caliper across the remaining covariates. The final adjacent-period population comprised 134 matched pairs. All post-matching |SMD| values were <0.10; the maximum was 0.063. * Continuous EuroSCORE II was not included in the propensity-score model and is shown as an additional balance diagnostic. ASA, American Society of Anesthesiologists Physical Status; eGFR, estimated glomerular filtration rate; Hb, hemoglobin; NYHA, New York Heart Association; SMD, standardized mean difference.

Table S4. Clinical and economic outcomes in the adjacent-period matched analysis of 2018 No-PBM controls and 2019 PBM patients.

Outcome	2019 PBM (n = 134)	2018 No-PBM (n = 134)	Matched Estimate (95% CI)	p-Value
Any RBC transfusion, n (%)	18 (13.4%)	54 (40.3%)	OR 0.28 (0.15–0.51)	<0.001
Transfusion of 1–2 RBC units, n (%)	16 (11.9%)	31 (23.1%)	OR 0.48 (0.26–0.91)	0.025
Transfusion of > 2 RBC units, n (%)	2 (1.5%)	23 (17.2%)	OR 0.09 (0.02–0.37)	<0.001
Total postoperative LOS, days, mean	7.02	9.21	MD −2.19 (−3.96 to −0.41)	—
Readmission within 12 months, n (%)	6 (4.5%)	8 (6.0%)	OR 0.75 (0.26–2.16)	0.590
Overall direct hospital cost, EUR, mean	13,656	19,544	MD −5888 (−10,707 to −1833)	—

Note: Binary outcomes were analyzed using conditional logistic regression. Continuous non-cost outcomes were analyzed using paired comparisons. Mean differences were calculated as 2019 PBM minus 2018 No-PBM; negative values indicate lower values in the PBM group. The 95% CI for the overall direct hospital cost difference was obtained by nonparametric bootstrap resampling of matched pairs; no p-value is reported for the economic comparison because uncertainty was quantified using the bootstrap CI. CI, confidence interval; LOS, length of stay; MD, mean difference; No-PBM, historical control cohort not managed through the structured Patient Blood Management pathway; OR, odds ratio; PBM, Patient Blood Management; RBC, red blood cell.

Table S5. Full coefficient estimates from the expanded individual-covariate and EuroSCORE-based logistic-regression models for RBC transfusion outcomes. (**A**) Any RBC transfusion. (**B**) 1–2 RBC units. (**C**) >2 RBC units.

Predictor	Comparison or Unit	β	SE	Wald z	Exp(β)	95% CI for Exp(β)	*p*-Value
(**A**)							
Primary Individual-Covariate Model							
Intercept	Model intercept	−0.4747	0.7906	−0.600	0.622	0.13–2.93	0.548
PBM pathway	PBM vs. No-PBM (reference)	−1.2194	0.1354	−9.003	0.295	0.23–0.39	<0.001
Age	Per 1-year increase	0.0010	0.0064	0.152	1.001	0.99–1.01	0.879
Male sex	Male vs. female (reference)	0.0344	0.1406	0.245	1.035	0.79–1.36	0.807
Baseline Hb	Per 1 g/dL increase	−0.0045	0.0422	−0.106	0.996	0.92–1.08	0.916
Isolated valve surgery	vs. CABG-containing procedures (reference)	0.0854	0.1619	0.527	1.089	0.79–1.50	0.598
Other non-CABG procedures	vs. CABG-containing procedures (reference)	0.1930	0.1703	1.134	1.213	0.87–1.69	0.257
NYHA III–IV	III–IV vs. I–II (reference)	0.1718	0.1539	1.117	1.187	0.88–1.61	0.264
ASA III–IV	III–IV vs. I–II (reference)	−0.0847	0.2947	−0.287	0.919	0.52–1.64	0.774
eGFR < 30 mL/min/1.73 m^2	vs. >60 mL/min/1.73 m^2 (reference)	−0.1798	0.4313	−0.417	0.835	0.36–1.95	0.677
eGFR 30–60 mL/min/1.73 m^2	vs. >60 mL/min/1.73 m^2 (reference)	0.3455	0.1812	1.907	1.413	0.99–2.01	0.056
Previous cardiac surgery	Yes vs. no (reference)	0.0935	0.4206	0.222	1.098	0.48–2.50	0.824
Diabetes mellitus	Yes vs. no (reference)	−0.1144	0.1442	−0.793	0.892	0.67–1.18	0.428
Preoperative anticoagulant use	Yes vs. no (reference)	−0.1526	0.1810	−0.843	0.858	0.60–1.22	0.399
Preoperative antiplatelet therapy	Yes vs. no (reference)	0.0091	0.1465	0.062	1.009	0.76–1.34	0.950
EuroSCORE-based sensitivity model							
Intercept	Model intercept	−0.0773	0.6395	−0.121	0.926	0.26–3.24	0.904
PBM pathway	PBM vs. No-PBM (reference)	−1.1261	0.1284	−8.773	0.324	0.25–0.42	<0.001
EuroSCORE II	Per 1 percentage-point increase	−0.0332	0.0363	−0.917	0.967	0.90–1.04	0.359
Baseline Hb	Per 1 g/dL increase	−0.0188	0.0405	−0.465	0.981	0.91–1.06	0.642
Isolated valve surgery	vs. CABG-containing procedures (reference)	0.0582	0.1558	0.374	1.060	0.78–1.44	0.708
Other non-CABG procedures	vs. CABG-containing procedures (reference)	0.1918	0.1626	1.180	1.211	0.88–1.67	0.238
ASA III–IV	III–IV vs. I–II (reference)	−0.1005	0.2912	−0.345	0.904	0.51–1.60	0.730
Preoperative anticoagulant use	Yes vs. no (reference)	−0.1313	0.1795	−0.731	0.877	0.62–1.25	0.464
Preoperative antiplatelet therapy	Yes vs. no (reference)	−0.0341	0.1421	−0.240	0.967	0.73–1.28	0.811
(**B**)							
Primary Individual-Covariate Model							
Intercept	Model intercept	−1.2587	0.8848	−1.423	0.284	0.05–1.61	0.155
PBM pathway	PBM vs. No-PBM (reference)	−0.7143	0.1486	−4.807	0.490	0.37–0.66	<0.001
Age	Per 1-year increase	−0.0003	0.0071	−0.040	1.000	0.99–1.01	0.968
Male sex	Male vs. female (reference)	−0.0329	0.1540	−0.214	0.968	0.72–1.31	0.831
Baseline Hb	Per 1 g/dL increase	−0.0197	0.0469	−0.421	0.980	0.89–1.07	0.674
Isolated valve surgery	vs. CABG-containing procedures (reference)	0.0588	0.1834	0.321	1.061	0.74–1.52	0.749
Other non-CABG procedures	vs. CABG-containing procedures (reference)	0.3632	0.1875	1.937	1.438	1.00–2.08	0.053
NYHA III–IV	III–IV vs. I–II (reference)	0.1465	0.1679	0.872	1.158	0.83–1.61	0.383
ASA III–IV	III–IV vs. I–II (reference)	0.2854	0.3469	0.823	1.330	0.67–2.63	0.411
eGFR < 30 mL/min/1.73 m^2	vs. >60 mL/min/1.73 m^2 (reference)	0.1814	0.4425	0.410	1.199	0.50–2.85	0.682
eGFR 30–60 mL/min/1.73 m^2	vs. >60 mL/min/1.73 m^2 (reference)	0.3276	0.1957	1.673	1.388	0.95–2.04	0.094
Previous cardiac surgery	Yes vs. no (reference)	0.1689	0.4390	0.385	1.184	0.50–2.80	0.701
Diabetes mellitus	Yes vs. no (reference)	0.0187	0.1592	0.117	1.019	0.75–1.39	0.907
Preoperative anticoagulant use	Yes vs. no (reference)	−0.1009	0.1981	−0.509	0.904	0.61–1.33	0.610
Preoperative antiplatelet therapy	Yes vs. no (reference)	−0.1254	0.1641	−0.764	0.882	0.64–1.22	0.445
EuroSCORE-Based Sensitivity Model							
Intercept	Model intercept	−0.8499	0.7216	−1.178	0.427	0.10–1.76	0.239
PBM pathway	PBM vs. No-PBM (reference)	−0.6357	0.1418	−4.484	0.530	0.40–0.70	<0.001
EuroSCORE II	Per 1 percentage-point increase	−0.0415	0.0420	−0.986	0.959	0.88–1.04	0.324
Baseline Hb	Per 1 g/dL increase	−0.0389	0.0452	−0.860	0.962	0.88–1.05	0.390
Isolated valve surgery	vs. CABG-containing procedures (reference)	0.0476	0.1767	0.270	1.049	0.74–1.48	0.787
Other non-CABG procedures	vs. CABG-containing procedures (reference)	0.3736	0.1791	2.086	1.453	1.02–2.06	0.037
ASA III–IV	III–IV vs. I–II (reference)	0.2389	0.3434	0.696	1.270	0.65–2.49	0.487
Preoperative anticoagulant use	Yes vs. no (reference)	−0.0862	0.1971	−0.437	0.917	0.62–1.35	0.662
Preoperative antiplatelet therapy	Yes vs. no (reference)	−0.1351	0.1593	−0.848	0.874	0.64–1.19	0.396
(**C**)							
Primary Individual-Covariate Model							
Intercept	Model intercept	−1.6308	1.2391	−1.316	0.196	0.02–2.22	0.188
PBM pathway	PBM vs. No-PBM (reference)	−1.9472	0.2649	−7.350	0.143	0.08–0.24	<0.001
Age	Per 1-year increase	0.0033	0.0102	0.322	1.003	0.98–1.02	0.748

Predictor	Comparison or Unit	β	SE	Wald z	Exp(β)	95% CI for Exp(β)	*p*-Value
Male sex	Male vs. female (reference)	0.1714	0.2391	0.717	1.187	0.74–1.90	0.473
Baseline Hb	Per 1 g/dL increase	0.0250	0.0666	0.375	1.025	0.90–1.17	0.708
Isolated valve surgery	vs. CABG-containing procedures (reference)	0.1014	0.2513	0.404	1.107	0.68–1.81	0.687
Other non-CABG procedures	vs. CABG-containing procedures (reference)	−0.3780	0.2956	−1.279	0.685	0.38–1.22	0.201
NYHA III–IV	III–IV vs. I–II (reference)	0.1553	0.2640	0.588	1.168	0.70–1.96	0.556
ASA III–IV	III–IV vs. I–II (reference)	−0.7494	0.4022	−1.863	0.473	0.21–1.04	0.062
eGFR < 30 mL/min/1.73 m²	vs. >60 mL/min/1.73 m² (reference)	−1.3292	1.0478	−1.269	0.265	0.03–2.06	0.205
eGFR 30–60 mL/min/1.73 m²	vs. >60 mL/min/1.73 m² (reference)	0.2004	0.3156	0.635	1.222	0.66–2.27	0.526
Previous cardiac surgery	Yes vs. no (reference)	−0.3262	1.0439	−0.312	0.722	0.09–5.58	0.755
Diabetes mellitus	Yes vs. no (reference)	−0.3537	0.2379	−1.487	0.702	0.44–1.12	0.137
Preoperative anticoagulant use	Yes vs. no (reference)	−0.1454	0.3069	−0.474	0.865	0.47–1.58	0.636
Preoperative antiplatelet therapy	Yes vs. no (reference)	0.3327	0.2373	1.402	1.395	0.88–2.22	0.161
EuroSCORE-Based Sensitivity Model							
Intercept	Model intercept	−1.5051	0.9670	−1.556	0.222	0.03–1.48	0.120
PBM pathway	PBM vs. No-PBM (reference)	−1.8628	0.2551	−7.301	0.155	0.09–0.26	<0.001
EuroSCORE II	Per 1-percentage-point increase	−0.0038	0.0578	−0.066	0.996	0.89–1.12	0.947
Baseline Hb	Per 1 g/dL increase	0.0317	0.0626	0.507	1.032	0.91–1.17	0.612
Isolated valve surgery	vs. CABG-containing procedures (reference)	0.0608	0.2429	0.250	1.063	0.66–1.71	0.802
Other non-CABG procedures	vs. CABG-containing procedures (reference)	−0.3759	0.2804	−1.341	0.687	0.40–1.19	0.180
ASA III–IV	III–IV vs. I–II (reference)	−0.6639	0.3936	−1.687	0.515	0.24–1.11	0.092
Preoperative anticoagulant use	Yes vs. no (reference)	−0.1172	0.3018	−0.388	0.889	0.49–1.61	0.698
Preoperative antiplatelet therapy	Yes vs. no (reference)	0.2218	0.2270	0.977	1.248	0.80–1.95	0.328

Note: The No-PBM cohort was the reference group for the PBM-pathway coefficient. CABG-containing procedures were the reference category for procedure type, and eGFR >60 mL/min/1.73 m² was the reference category for renal function. The primary individual-covariate models included PBM pathway, age, sex, baseline Hb, procedure type, NYHA class, ASA physical status, eGFR category, previous cardiac surgery, diabetes mellitus, preoperative anticoagulant use, and antiplatelet therapy. The EuroSCORE-based sensitivity models included PBM pathway, continuous EuroSCORE II, baseline Hb, procedure type, ASA physical status, anticoagulant use, and antiplatelet therapy. All models included 1374 patients. The intercept is provided for completeness; exp(β) for predictor rows is the adjusted odds ratio. *p*-values are two-sided Wald tests. β, logistic-regression coefficient; ASA, American Society of Anesthesiologists; CABG, coronary artery bypass grafting; CI, confidence interval; eGFR, estimated glomerular filtration rate; Hb, hemoglobin; NYHA, New York Heart Association; PBM, Patient Blood Management; RBC, red blood cell; SE, standard error.

Table S6. Event-to-variable ratios, convergence, influence, multicollinearity, and internal-validation diagnostics for the expanded logistic-regression models. (**A**) Model size, sparsity, convergence, and collinearity. (**B**) Likelihood, fitted-probability, discrimination, and overall-error diagnostics. (**C**) Additional stability and bootstrap diagnostics for transfusion of >2 RBC units (primary individual-covariate model).

(A)												
**Outcome**	**Model**	***n***	**Events**	**Non-Events**	**Predictor Variables**	**Predictor Parameters**	**Events per Variable**	**Events per Parameter**	**Convergence**	**Separation**	**Finite Estimates**	**Maximum VIF**
Any RBC transfusion	Individual-covariate	1374	371	1003	12	14	30.9	26.5	Yes (6 iterations)	No separation detected	Yes	1.568
Any RBC transfusion	EuroSCORE-based	1374	371	1003	7	8	53.0	46.4	Yes (6 iterations)	No separation detected	Yes	1.438
1–2 RBC units	Individual-covariate	1374	259	1115	12	14	21.6	18.5	Yes (6 iterations)	No separation detected	Yes	1.568
1–2 RBC units	EuroSCORE-based	1374	259	1115	7	8	37.0	32.4	Yes (6 iterations)	No separation detected	Yes	1.438
>2 RBC units	Individual-covariate	1374	112	1262	12	14	9.3	8.0	Yes (8 iterations)	No separation detected	Yes	1.568

>2 RBC units	EuroSCORE-based	1374	112	1262	7	8	16.0	14.0	Yes (8 iterations)	No separation detected	Yes	1.438

(B)

Outcome	Model	Fitted Probability Range	Likelihood-Ratio χ^2 (df)	Model *p*-Value	Apparent AUC	Apparent Brier Score	Comment
Any RBC transfusion	Individual-covariate	0.1182–0.5596	91.48 (14)	<0.001	0.660	0.1841	Apparent (in-sample) diagnostic
Any RBC transfusion	EuroSCORE-based	0.0433–0.4532	86.10 (8)	<0.001	0.652	0.1849	Apparent (in-sample) diagnostic
1–2 RBC units	Individual-covariate	0.0815–0.3860	30.15 (14)	0.007	0.606	0.1496	Apparent (in-sample) diagnostic
1–2 RBC units	EuroSCORE-based	0.0222–0.3381	27.54 (8)	<0.001	0.606	0.1499	Apparent (in-sample) diagnostic
>2 RBC units	Individual-covariate	0.0030–0.3069	85.87 (14)	<0.001	0.753	0.0697	Apparent (in-sample) diagnostic
>2 RBC units	EuroSCORE-based	0.0147–0.2736	79.37 (8)	<0.001	0.737	0.0706	Apparent (in-sample) diagnostic

(C)

Diagnostic	Result	Interpretation or detail
Outcome events	112	20/764 PBM patients and 92/610 No-PBM controls
Predictor variables/parameters	12/14	Intercept excluded from both counts
Events per variable/parameter	9.3/8.0	Lowest ratio among the three transfusion models
Conventional maximum-likelihood PBM estimate	OR 0.143 (0.085–0.240)	Two-sided Wald $p < 0.001$
Leave-one-out PBM OR range	0.134–0.147	Model refitted after omission of each patient in turn; 1374/1374 fits converged
Firth bias-reduced PBM estimate	OR 0.148 (0.087–0.243)	Profile penalized-likelihood CI; penalized likelihood-ratio $p < 0.001$
Bootstrap resamples	1000	All 1000 bootstrap resamples converged successfully
AUC	0.7528 apparent	0.718 optimism-corrected
Brier score	0.0697 apparent	0.0722 optimism-corrected
Calibration-in-the-large	0.0000 apparent	0.006 optimism-corrected; slope fixed at 1
Joint calibration intercept	0.0000 apparent	−0.5867 optimism-corrected; used with corrected slope in Figure S3
Calibration slope	1.0000 apparent	0.720 optimism-corrected
Fitted probability range	0.0030–0.3069	Conventional primary model
Multicollinearity	Maximum VIF 1.568	VIFs calculated with the intercept included in the auxiliary regressions
Separation	No separation detected	Maximum-likelihood model converged with finite coefficients
Overall interpretation	Some optimism detected	The corrected slope below 1 indicates some overfitting; the direction of the PBM association remained stable across conventional, Firth, PSM, and IPTW analyses.

Note: Predictor-variable counts treat each clinically named predictor as one variable, whereas predictor-parameter counts reflect the additional indicator coefficients required for three-level procedure type and eGFR. The intercept was excluded from both counts. Separation was assessed using a linear-programming feasibility diagnostic; no complete or quasi-complete separation was detected. VIFs exclude the intercept as a reported predictor but were calculated from auxiliary regressions containing an intercept. Bootstrap optimism correction used 1000 resamples. Calibration-in-the-large was estimated with the original linear predictor entered as an offset (slope fixed at 1); the joint calibration intercept and slope were estimated together. AUC and calibration statistics are internal regression-model diagnostics and do not constitute development or external validation of a clinical prediction model. AUC, area under the receiver-operating-characteristic curve; PBM, Patient Blood Management; VIF, variance-inflation factor.

Table S7. Sensitivity analyses of overall direct hospital cost.

Analysis	PBM Mean Cost, EUR	No-PBM Mean Cost, EUR	Effect Estimate (95% CI Where Applicable)
Base-case unadjusted complete cohort	15,632.60	20,588.38	MD −4955.78 (−7008.91 to −3039.86)
Covariate-adjusted Gamma GLM with log link	15,544 *	20,739 *	Cost ratio 0.75 (0.68–0.82); marginal MD −5196 (−6965 to −3485)
Excluding readmission costs	—	—	MD −4436 (−6182 to −2792)
Individual unit-cost one-way analyses (±20%)	—	—	All scenarios retained a negative PBM minus No-PBM mean difference
Combined conservative unit-cost scenario †	—	—	MD −3928

Combined favorable unit-cost scenario ‡	—	—	MD −5984
Inverse-Gaussian GLM with log link	—	—	Cost ratio 0.75 (0.68–0.82)
Propensity-score-matched population	14,850	21,051	MD −6201 (−8481 to −4195)
Stabilized IPTW population	15,457	20,351	MD −4894 (−6735 to −3206)
Adjacent-period matched population (2019 vs. 2018)	13,656	19,544	MD −5888 (−10,707 to −1833)

Note: All costs are expressed in 2024 euros. Mean differences (MD) are calculated as PBM minus No-PBM; negative values indicate lower mean cost under PBM management. Bootstrap confidence intervals used 1000 resamples. * Adjusted marginal means from recycled predictions after the Gamma generalized linear model. † Conservative scenario: FCM and ESA unit costs increased by 20%, whereas RBC, T-SICU, and TSU unit costs were reduced by 20%. ‡ Favorable scenario: FCM and ESA unit costs reduced by 20%, whereas RBC, T-SICU, and TSU unit costs were increased by 20%. CI, confidence interval; ESA, erythropoiesis-stimulating agent; FCM, ferric carboxymaltose; GLM, generalized linear model; IPTW, inverse probability of treatment weighting; No-PBM, historical control cohort not managed through the structured Patient Blood Management pathway; PBM, Patient Blood Management; RBC, red blood cell; T-SICU, thoracic surgery intensive care unit; TSU, thoracic surgery unit.

Supplementary Table S8. Interval-specific postoperative safety events and descriptive event counts among FCM-treated and ESA-treated patients.

Panel A. Interval-specific postoperative safety events in the complete PBM and No-PBM cohorts

Safety outcome	PBM ≤30 days (n = 764)	No-PBM ≤30 days (n = 610)	p-value	PBM >30 days to 12 months (n = 764)	No-PBM >30 days to 12 months (n = 610)	p-value
Myocardial infarction	0 (0.00%)	2 (0.33%)	0.197	1 (0.13%)	0 (0.00%)	1.000
Stroke	4 (0.52%)	2 (0.33%)	0.699	1 (0.13%)	3 (0.49%)	0.328
Documented thromboembolism	0 (0.00%)	1 (0.16%)	0.444	0 (0.00%)	1 (0.16%)	0.444
Infection	25 (3.27%)	12 (1.97%)	0.179	1 (0.13%)	13 (2.13%)	<0.001
Re-exploration for bleeding or cardiac tamponade	9 (1.18%)	5 (0.82%)	0.596	0 (0.00%)	0 (0.00%)	—
Decompensated heart failure	0 (0.00%)	7 (1.15%)	0.003	1 (0.13%)	4 (0.66%)	0.178

Panel B. Cumulative 12-month postoperative events among FCM-treated and ESA-treated PBM patients

Safety outcome	FCM-treated patients (n = 355)	ESA-treated patients (n = 69)
Myocardial infarction	0 (0.00%)	0 (0.00%)
Stroke	3 (0.85%)	0 (0.00%)
Documented thromboembolism	0 (0.00%)	0 (0.00%)
Infection	11 (3.10%)	1 (1.45%)
Re-exploration for bleeding or cardiac tamponade	7 (1.97%)	3 (4.35%)
Decompensated heart failure	1 (0.28%)	0 (0.00%)

Note: Data are presented as n (%). In Panel A, the two follow-up intervals are non-overlapping; events occurring within 30 days and events occurring from >30 days to 12 months after surgery are shown separately. Between-cohort comparisons used two-sided Fisher exact tests. No adjustment for multiple comparisons was applied because these analyses were exploratory. A dash indicates that no formal comparison was performed because neither cohort experienced the event during that interval. Panel B presents cumulative 12-month event counts among PBM patients who received FCM or ESA. These exposure groups were not mutually exclusive: 66 patients received both FCM and ESA. No formal comparisons were performed between treatment-exposure groups because treatment was clinically determined and the groups overlapped. Events were identified retrospectively from routine clinical records, were not independently adjudicated, and should not be interpreted as adverse reactions causally attributed to FCM or ESA. Serum phosphate was not routinely measured after FCM, and structured data on acute infusion or hypersensitivity reactions, extravasation, treatment-emergent hypertension, treatment discontinuation, and other treatment-specific adverse effects were not systematically available and are therefore not shown. ESA, erythropoiesis-stimulating agent; FCM, ferric carboxymaltose; No-PBM, historical control cohort not managed through the structured Patient Blood Management pathway; PBM, Patient Blood Management.
